# Supplementary material for: Variation in ubiquitin system genes creates substrate-specific effects on proteasomal protein degradation
Source: eLife. 2022 Oct 11;11:e79570. doi: 10.7554/eLife.79570 (PMC9634822; doi:10.7554/eLife.79570)
Supplement: Supplementary file 1. — The plots show the loess-smoothed RM allele frequency difference (high UPS activity pool minus low UPS activity pool) and LOD score traces for the 20 N-degrons. QTLs are marked with asterisks, which are colored by biological replicate. [file elife-79570-supp1.pdf]

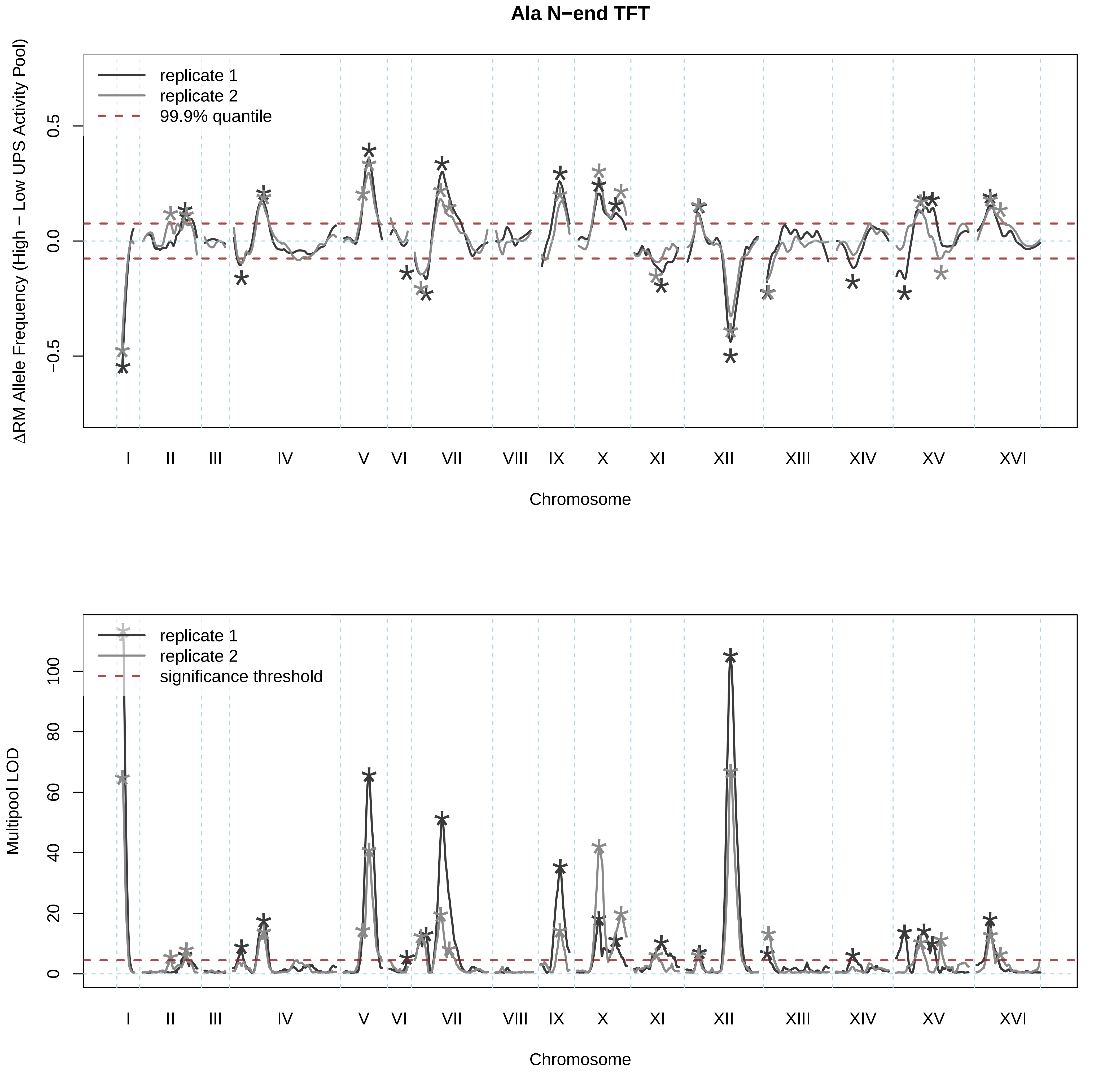

Ala N−end TFT
replicate 1
replicate 2
99.9% quantile
ΔRM Allele Frequency (High − Low UPS Activity Pool)
0.5
0.0
−0.5
I
II
III
IV
V
VI
VII
VIII
IX
X
XI
XII
XIII
XIV
XV
XVI
Chromosome
replicate 1
replicate 2
significance threshold
Multipool LOD
100
80
60
40
20
0
I
II
III
IV
V
VI
VII
VIII
IX
X
XI
XII
XIII
XIV
XV
XVI
Chromosome

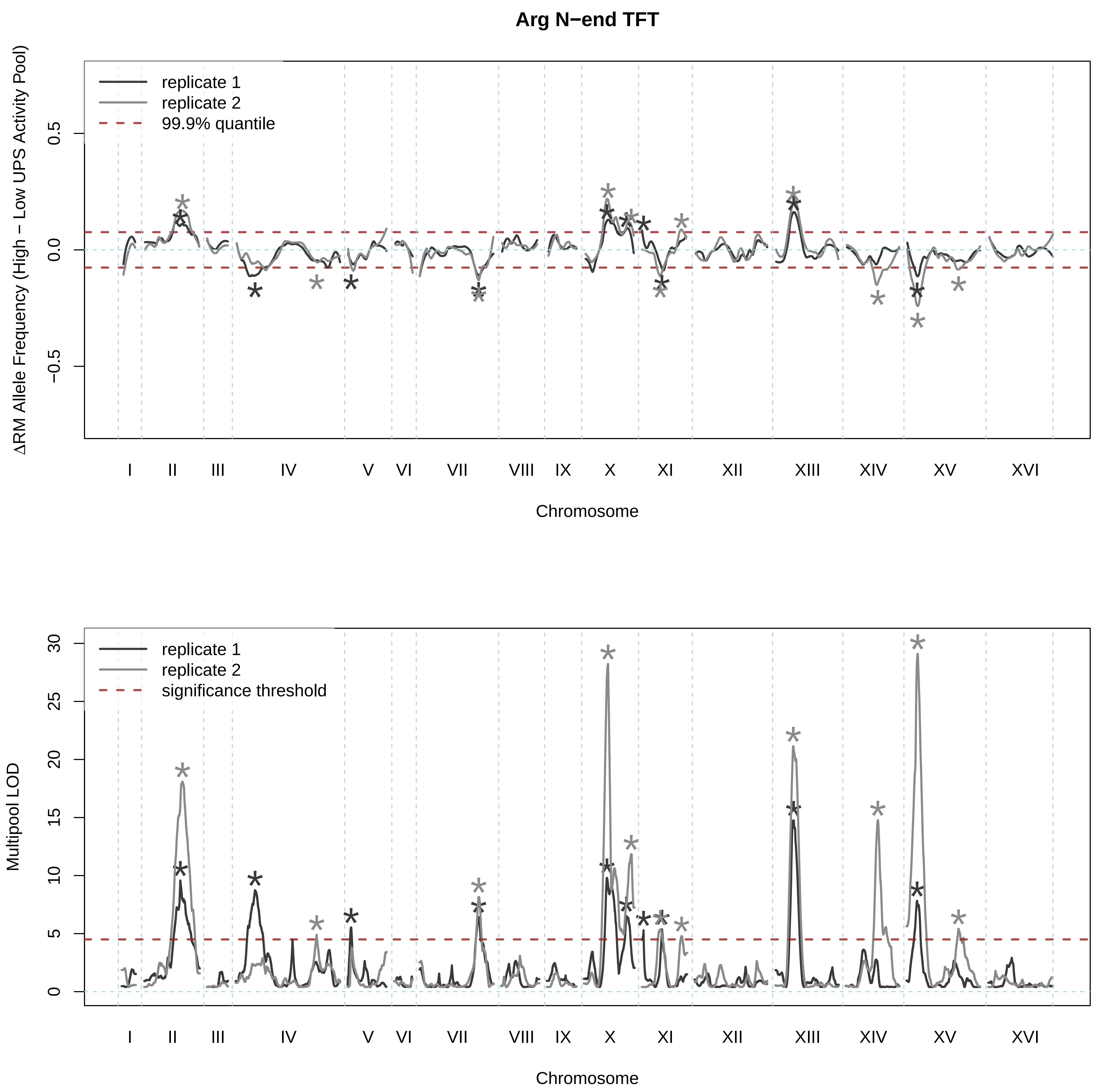

Arg N−end TFT
replicate 1
replicate 2
99.9% quantile
ΔRM Allele Frequency (High − Low UPS Activity Pool)
0.5
0.0
−0.5
I
II
III
IV
V
VI
VII
VIII
IX
X
XI
XII
XIII
XIV
XV
XVI
Chromosome
replicate 1
replicate 2
significance threshold
Multipool LOD
0
5
10
15
20
25
30
Chromosome

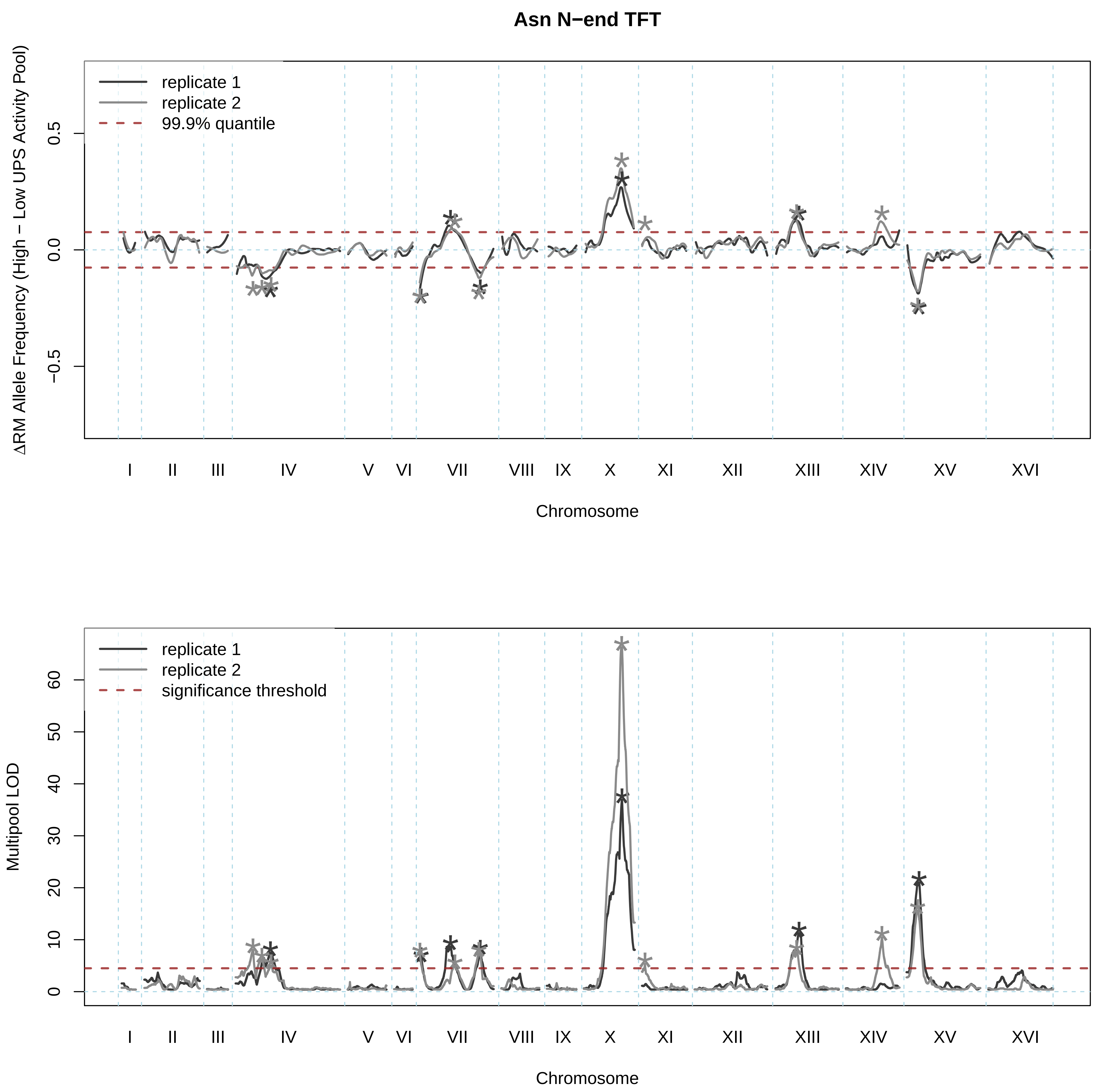

Asn N−end TFT
replicate 1
replicate 2
99.9% quantile
ΔRM Allele Frequency (High − Low UPS Activity Pool)
0.5
0.0
−0.5
I
II
III
IV
V
VI
VII
VIII
IX
X
XI
XII
XIII
XIV
XV
XVI
Chromosome
replicate 1
replicate 2
significance threshold
Multipool LOD
60
50
40
30
20
10
0
I
II
III
IV
V
VI
VII
VIII
IX
X
XI
XII
XIII
XIV
XV
XVI
Chromosome

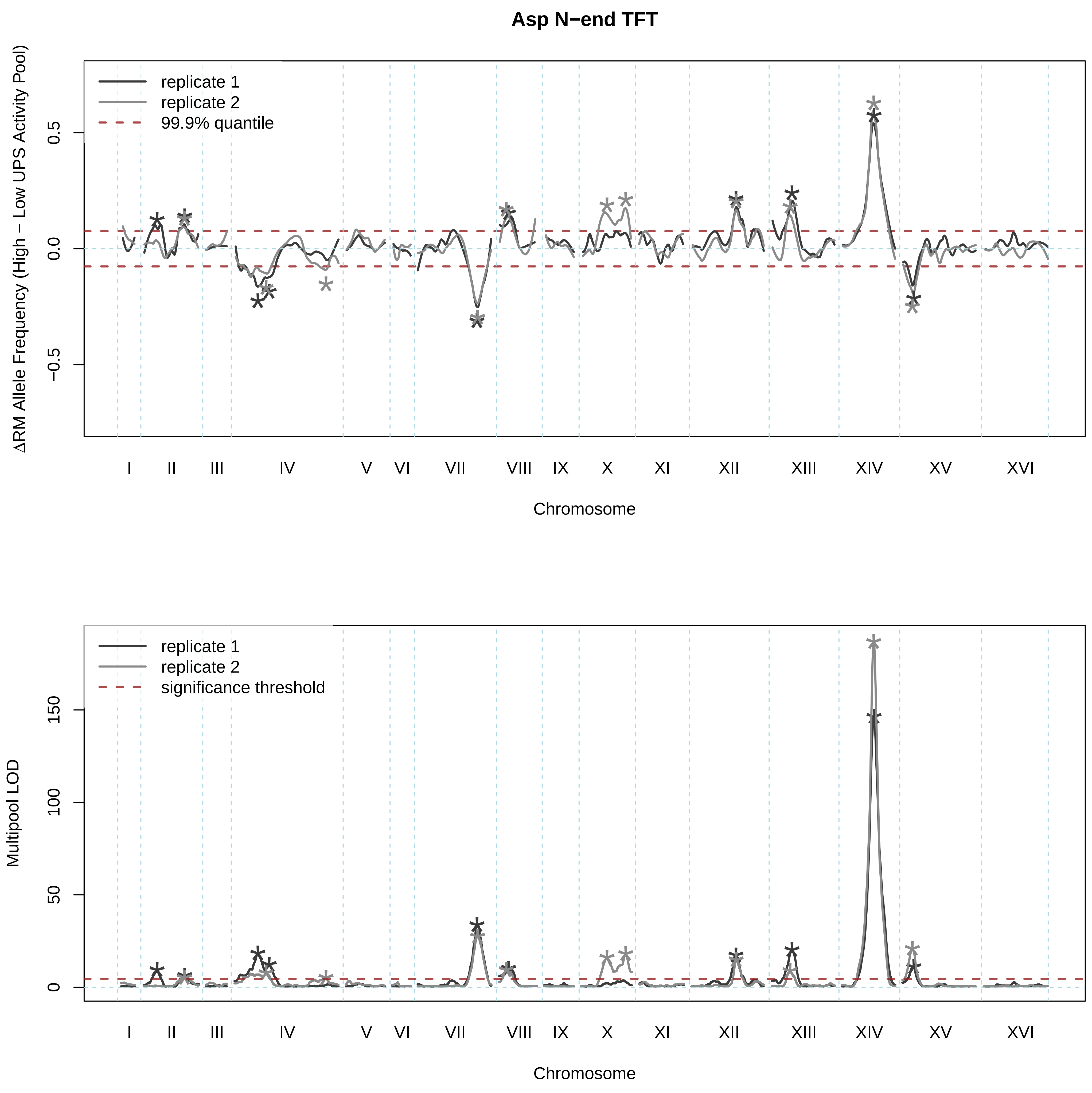

Asp N−end TFT
replicate 1
replicate 2
99.9% quantile
ΔRM Allele Frequency (High − Low UPS Activity Pool)
0.5
0.0
−0.5
I
II
III
IV
V
VI
VII
VIII
IX
X
XI
XII
XIII
XIV
XV
XVI
Chromosome
replicate 1
replicate 2
significance threshold
Multipool LOD
150
100
50
0
I
II
III
IV
V
VI
VII
VIII
IX
X
XI
XII
XIII
XIV
XV
XVI
Chromosome

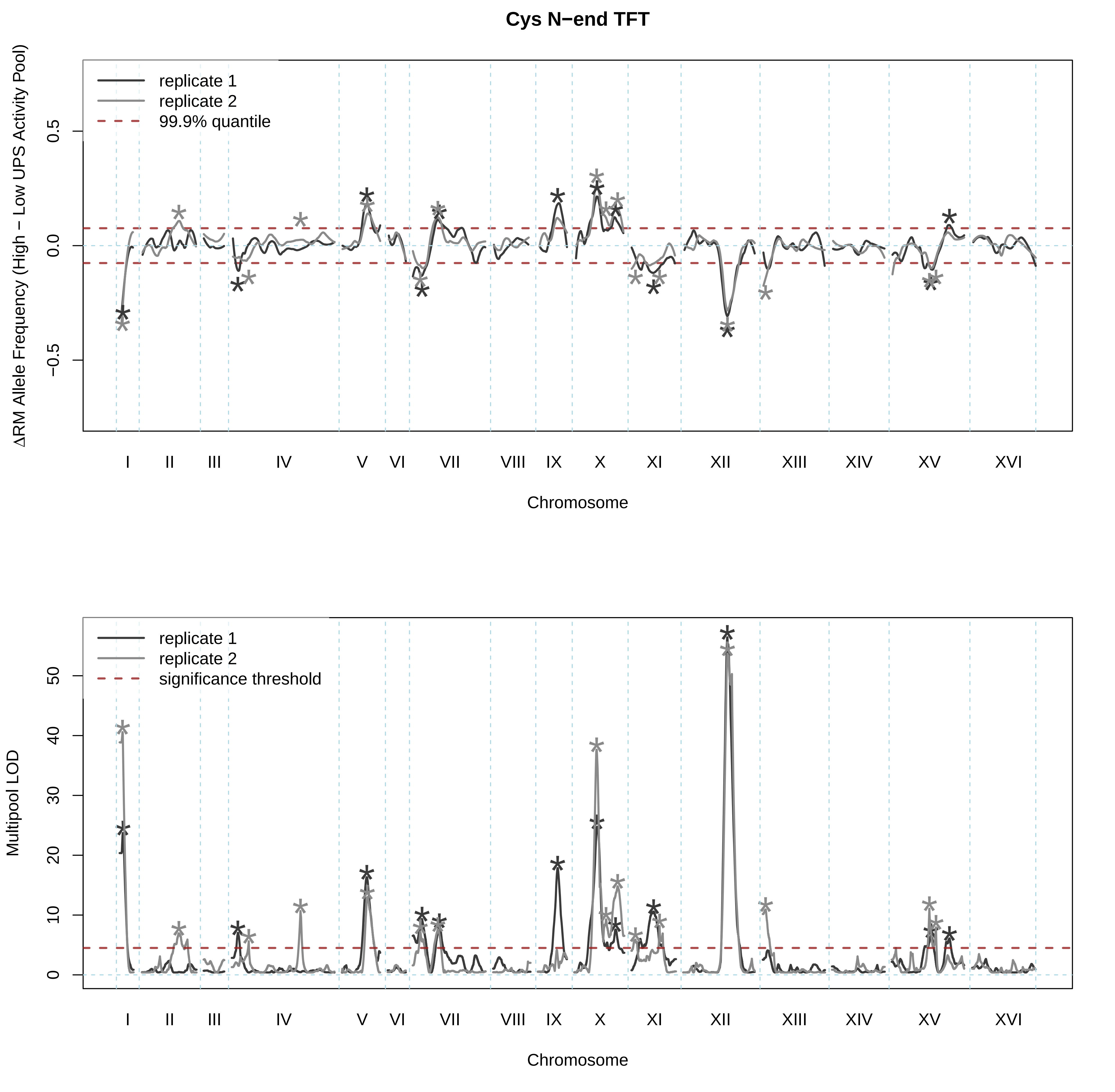

Cys N−end TFT
replicate 1
replicate 2
99.9% quantile
ΔRM Allele Frequency (High − Low UPS Activity Pool)
0.5
0.0
−0.5
I
II
III
IV
V
VI
VII
VIII
IX
X
XI
XII
XIII
XIV
XV
XVI
Chromosome
replicate 1
replicate 2
significance threshold
Multipool LOD
0
10
20
30
40
50
I
II
III
IV
V
VI
VII
VIII
IX
X
XI
XII
XIII
XIV
XV
XVI
Chromosome

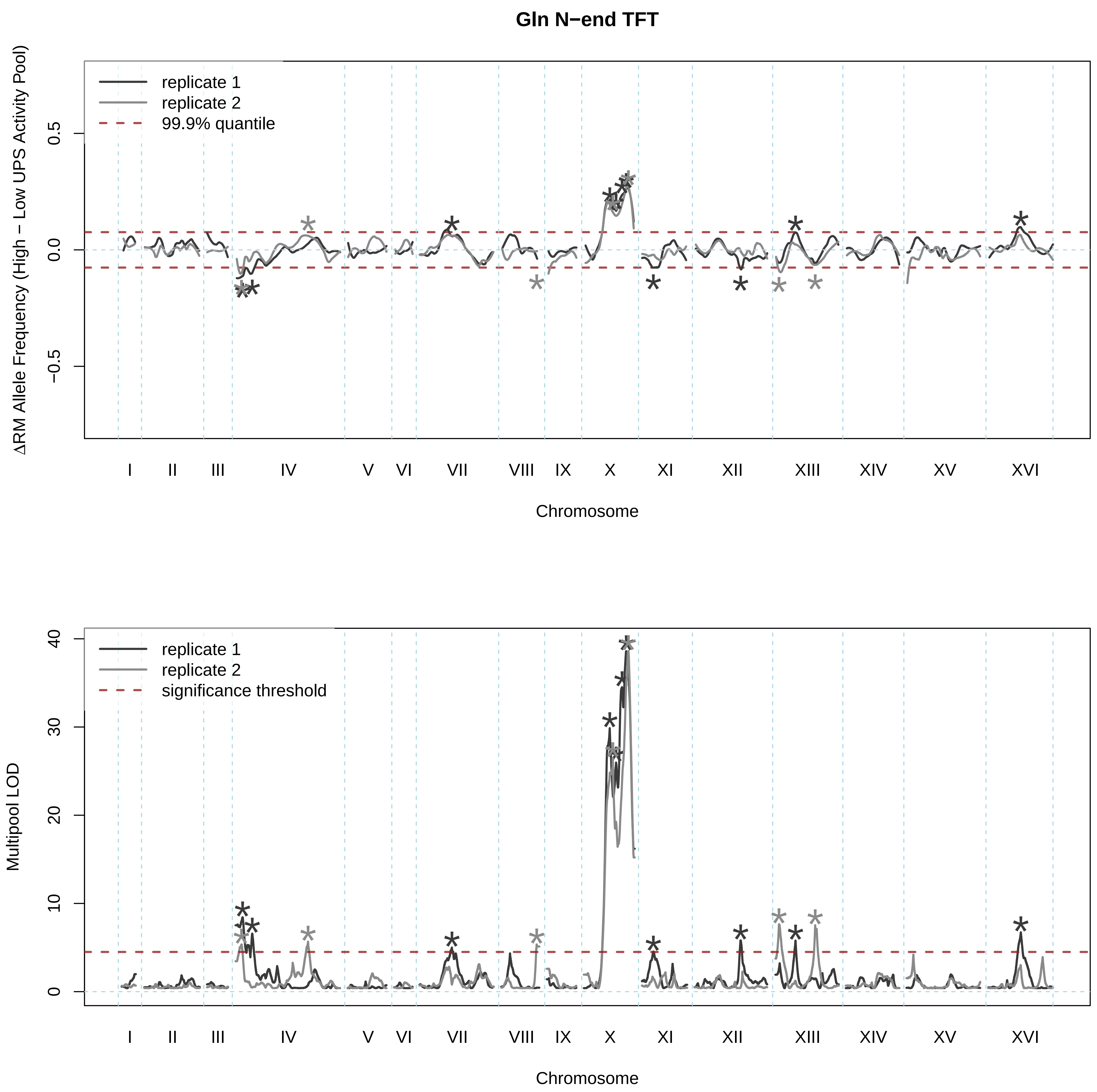

Gln N−end TFT
replicate 1
replicate 2
99.9% quantile
ΔRM Allele Frequency (High − Low UPS Activity Pool)
0.5
0.0
−0.5
I
II
III
IV
V
VI
VII
VIII
IX
X
XI
XII
XIII
XIV
XV
XVI
Chromosome
replicate 1
replicate 2
significance threshold
Multipool LOD
40
30
20
10
0
I
II
III
IV
V
VI
VII
VIII
IX
X
XI
XII
XIII
XIV
XV
XVI
Chromosome

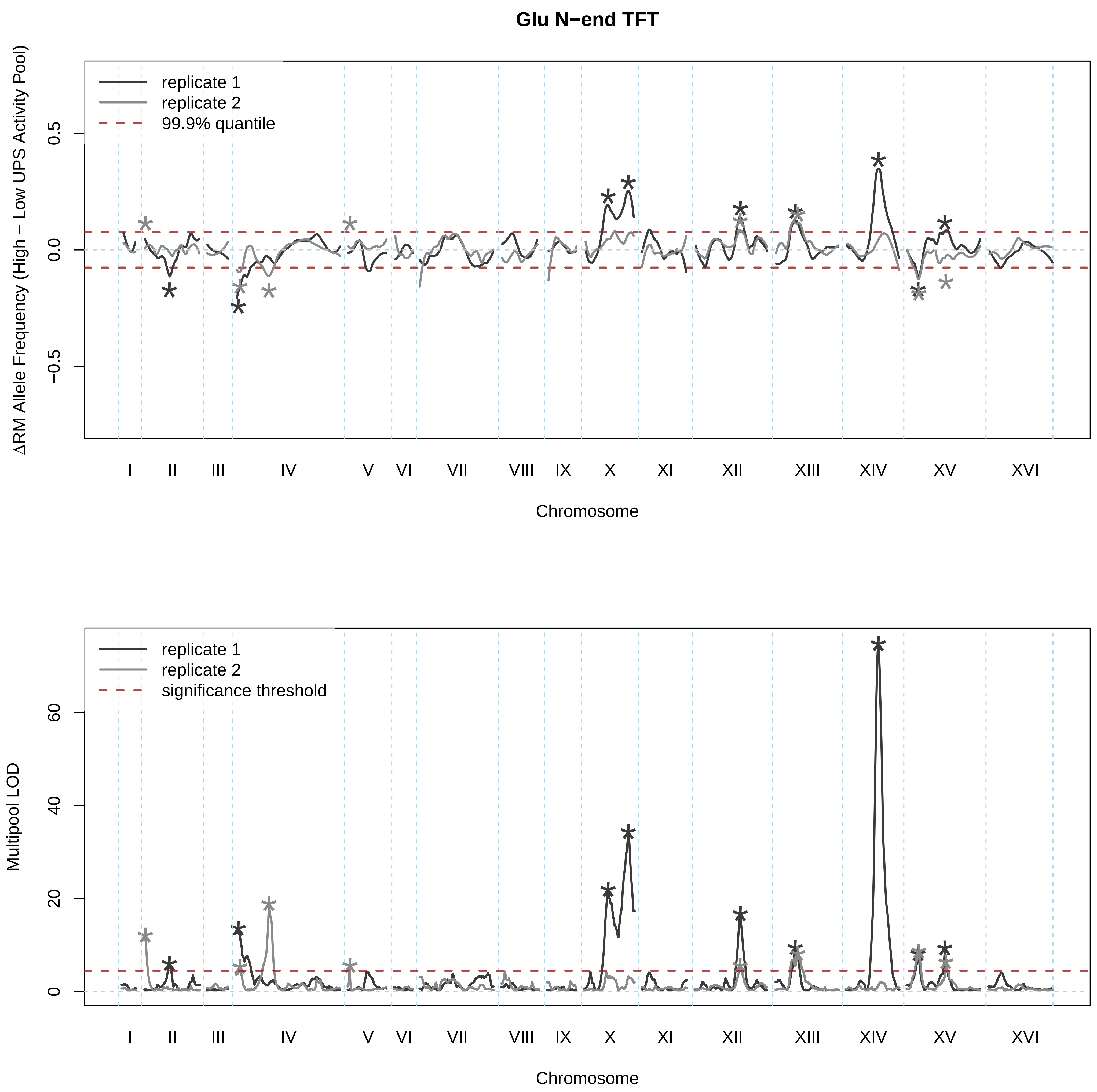

Glu N−end TFT
replicate 1
replicate 2
99.9% quantile
ΔRM Allele Frequency (High − Low UPS Activity Pool)
0.5
0.0
−0.5
I
II
III
IV
V
VI
VII
VIII
IX
X
XI
XII
XIII
XIV
XV
XVI
Chromosome
replicate 1
replicate 2
significance threshold
Multipool LOD
0
20
40
60
Chromosome

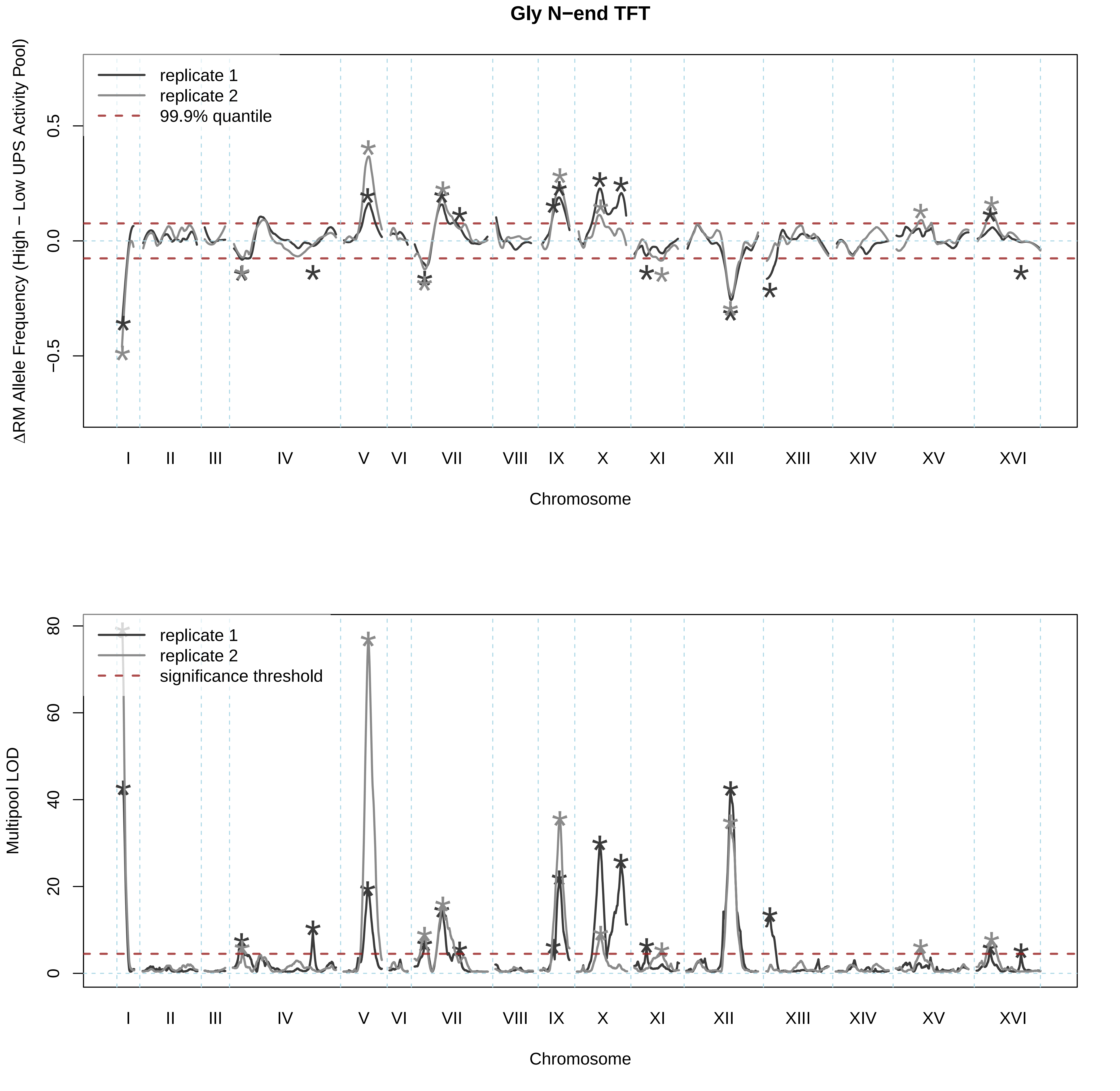

Gly N−end TFT
replicate 1
replicate 2
99.9% quantile
ΔRM Allele Frequency (High − Low UPS Activity Pool)
0.5
0.0
−0.5
I
II
III
IV
V
VI
VII
VIII
IX
X
XI
XII
XIII
XIV
XV
XVI
Chromosome
replicate 1
replicate 2
significance threshold
Multipool LOD
80
60
40
20
0
I
II
III
IV
V
VI
VII
VIII
IX
X
XI
XII
XIII
XIV
XV
XVI
Chromosome

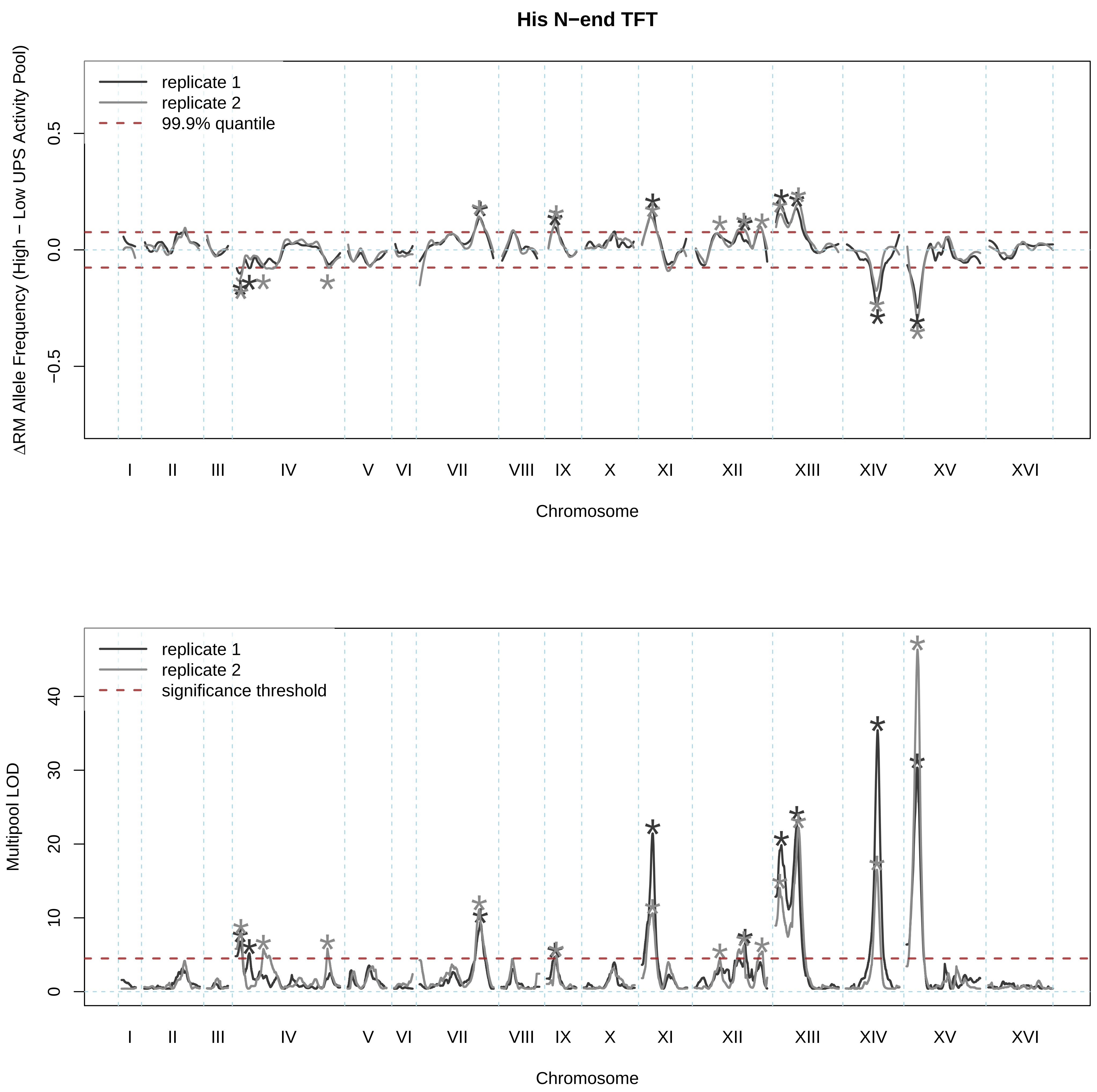

His N−end TFT
replicate 1
replicate 2
99.9% quantile
ΔRM Allele Frequency (High − Low UPS Activity Pool)
0.5
0.0
−0.5
I
II
III
IV
V
VI
VII
VIII
IX
X
XI
XII
XIII
XIV
XV
XVI
Chromosome
replicate 1
replicate 2
significance threshold
Multipool LOD
40
30
20
10
0
I
II
III
IV
V
VI
VII
VIII
IX
X
XI
XII
XIII
XIV
XV
XVI
Chromosome

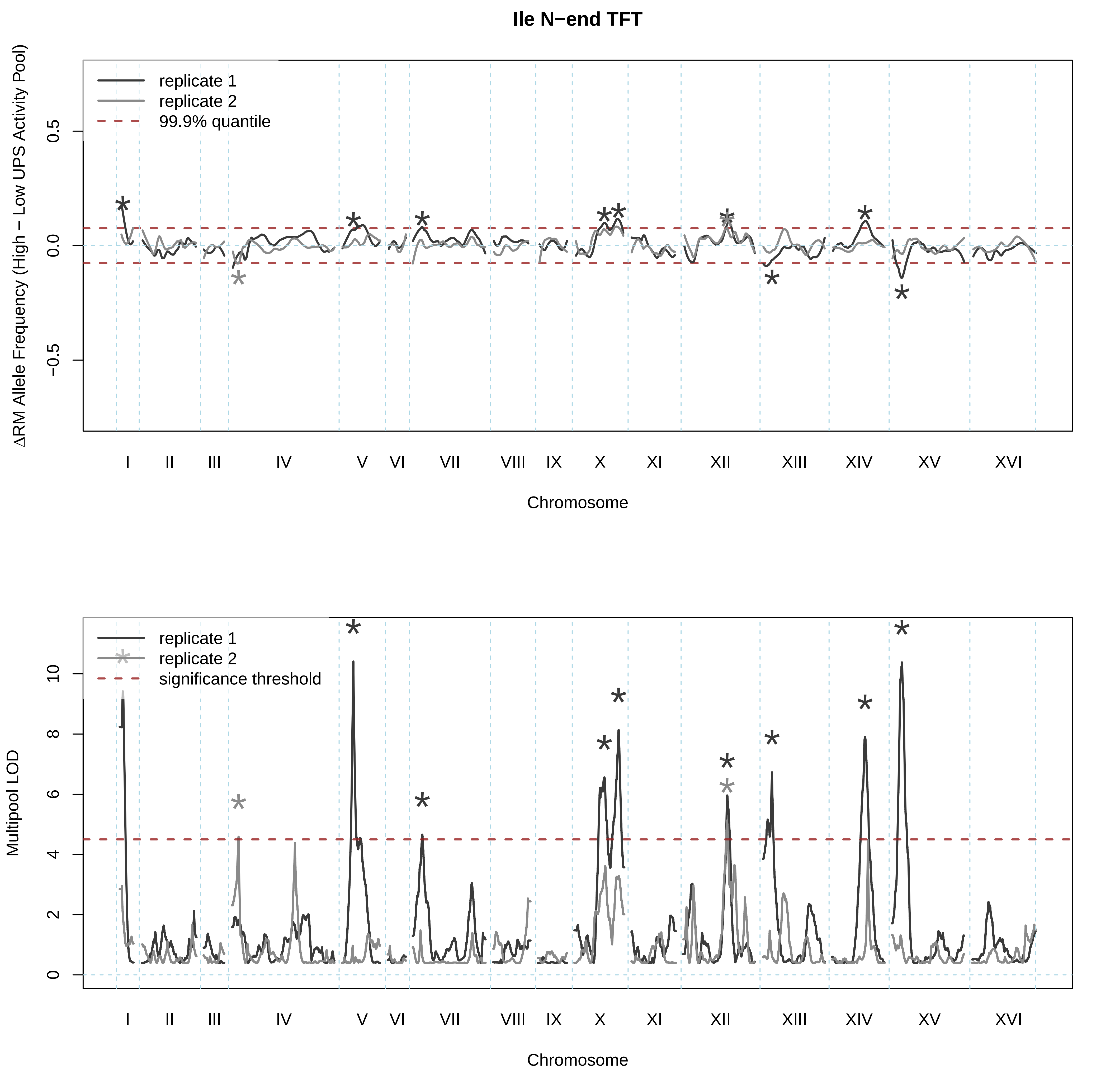

Ile N−end TFT
replicate 1
replicate 2
99.9% quantile
ΔRM Allele Frequency (High − Low UPS Activity Pool)
0.5
0.0
−0.5
I
II
III
IV
V
VI
VII
VIII
IX
X
XI
XII
XIII
XIV
XV
XVI
Chromosome
replicate 1
replicate 2
significance threshold
Multipool LOD
0
2
4
6
8
10
I
II
III
IV
V
VI
VII
VIII
IX
X
XI
XII
XIII
XIV
XV
XVI
Chromosome

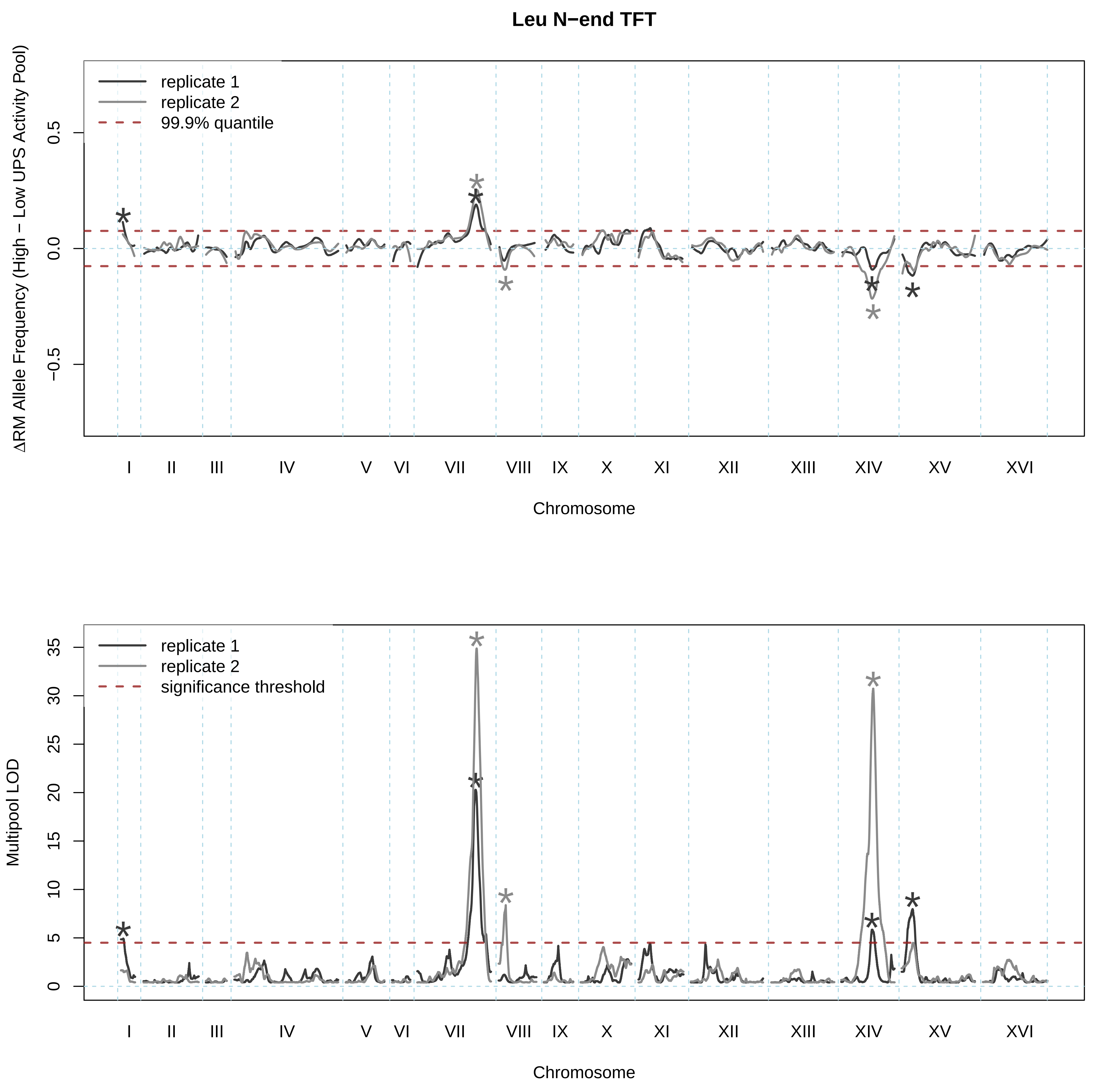

Leu N−end TFT
replicate 1
replicate 2
99.9% quantile
ΔRM Allele Frequency (High − Low UPS Activity Pool)
0.5
0.0
−0.5
I
II
III
IV
V
VI
VII
VIII
IX
X
XI
XII
XIII
XIV
XV
XVI
Chromosome
replicate 1
replicate 2
significance threshold
Multipool LOD
35
30
25
20
15
10
5
0
I
II
III
IV
V
VI
VII
VIII
IX
X
XI
XII
XIII
XIV
XV
XVI
Chromosome

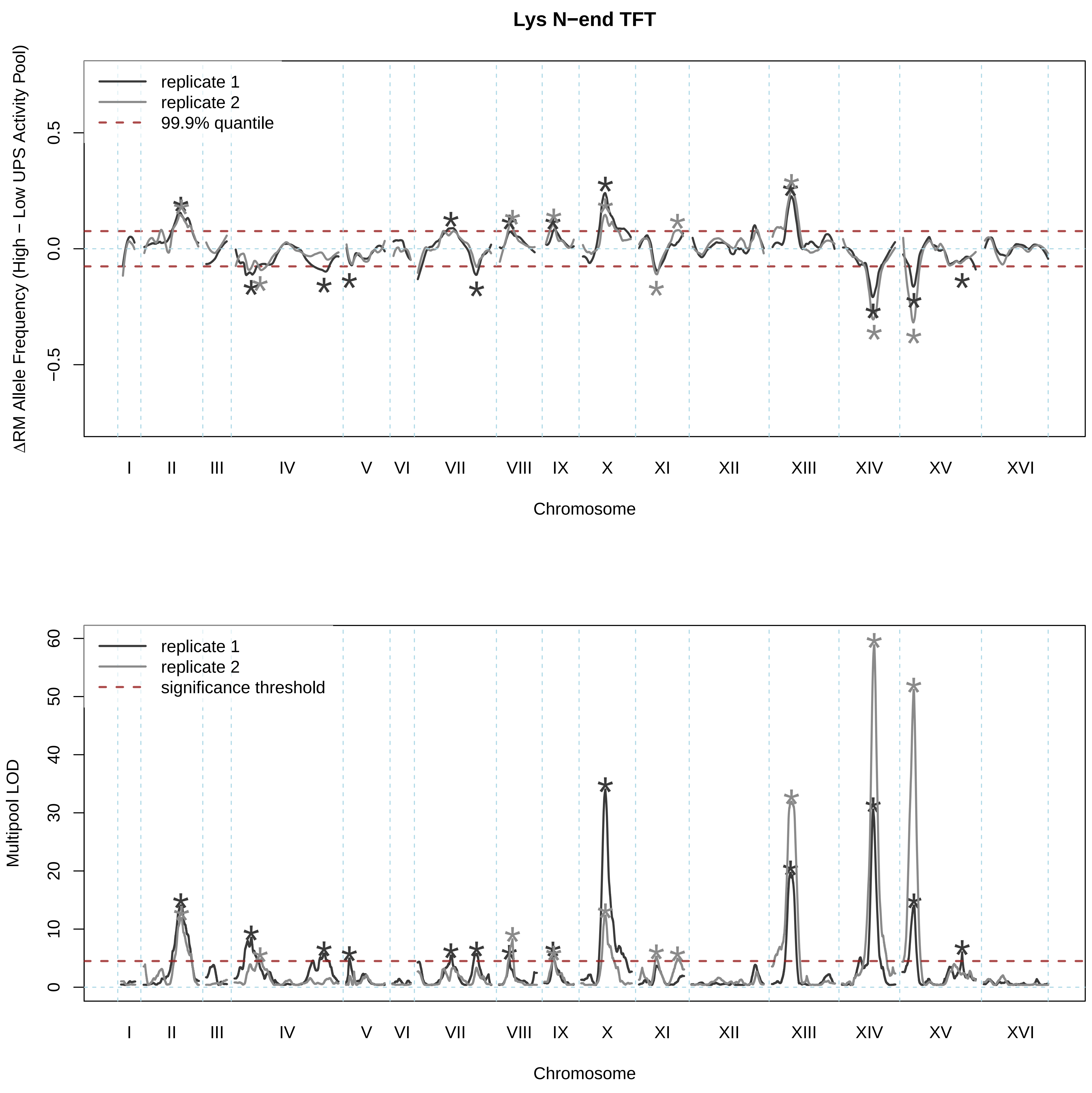

Lys N−end TFT
replicate 1
replicate 2
99.9% quantile
ΔRM Allele Frequency (High − Low UPS Activity Pool)
0.5
0.0
−0.5
I
II
III
IV
V
VI
VII
VIII
IX
X
XI
XII
XIII
XIV
XV
XVI
Chromosome
replicate 1
replicate 2
significance threshold
Multipool LOD
60
50
40
30
20
10
0
I
II
III
IV
V
VI
VII
VIII
IX
X
XI
XII
XIII
XIV
XV
XVI
Chromosome

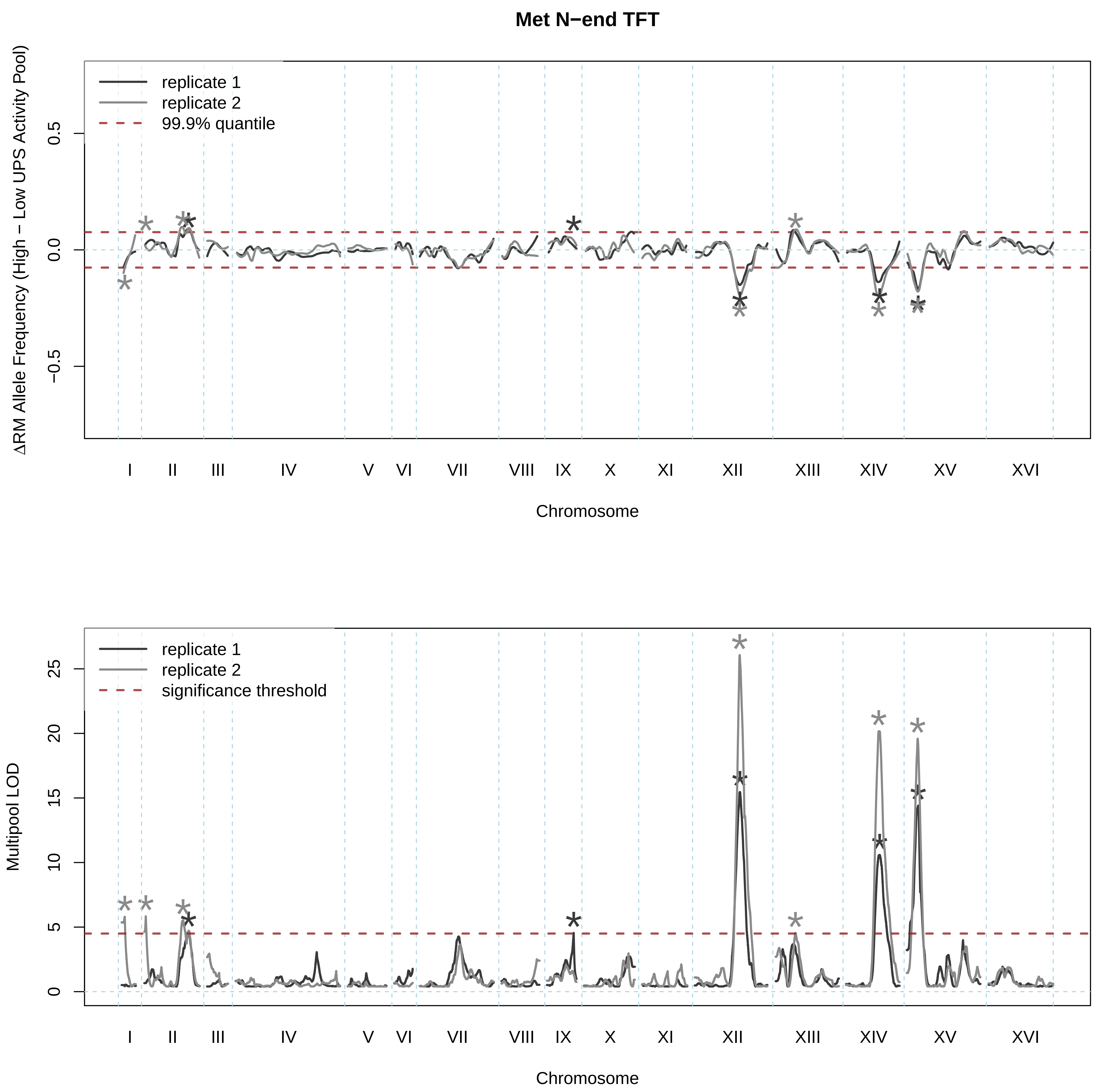

Met N−end TFT
replicate 1
replicate 2
99.9% quantile
ΔRM Allele Frequency (High − Low UPS Activity Pool)
0.5
0.0
−0.5
I
II
III
IV
V
VI
VII
VIII
IX
X
XI
XII
XIII
XIV
XV
XVI
Chromosome
replicate 1
replicate 2
significance threshold
Multipool LOD
25
20
15
10
5
0
I
II
III
IV
V
VI
VII
VIII
IX
X
XI
XII
XIII
XIV
XV
XVI
Chromosome

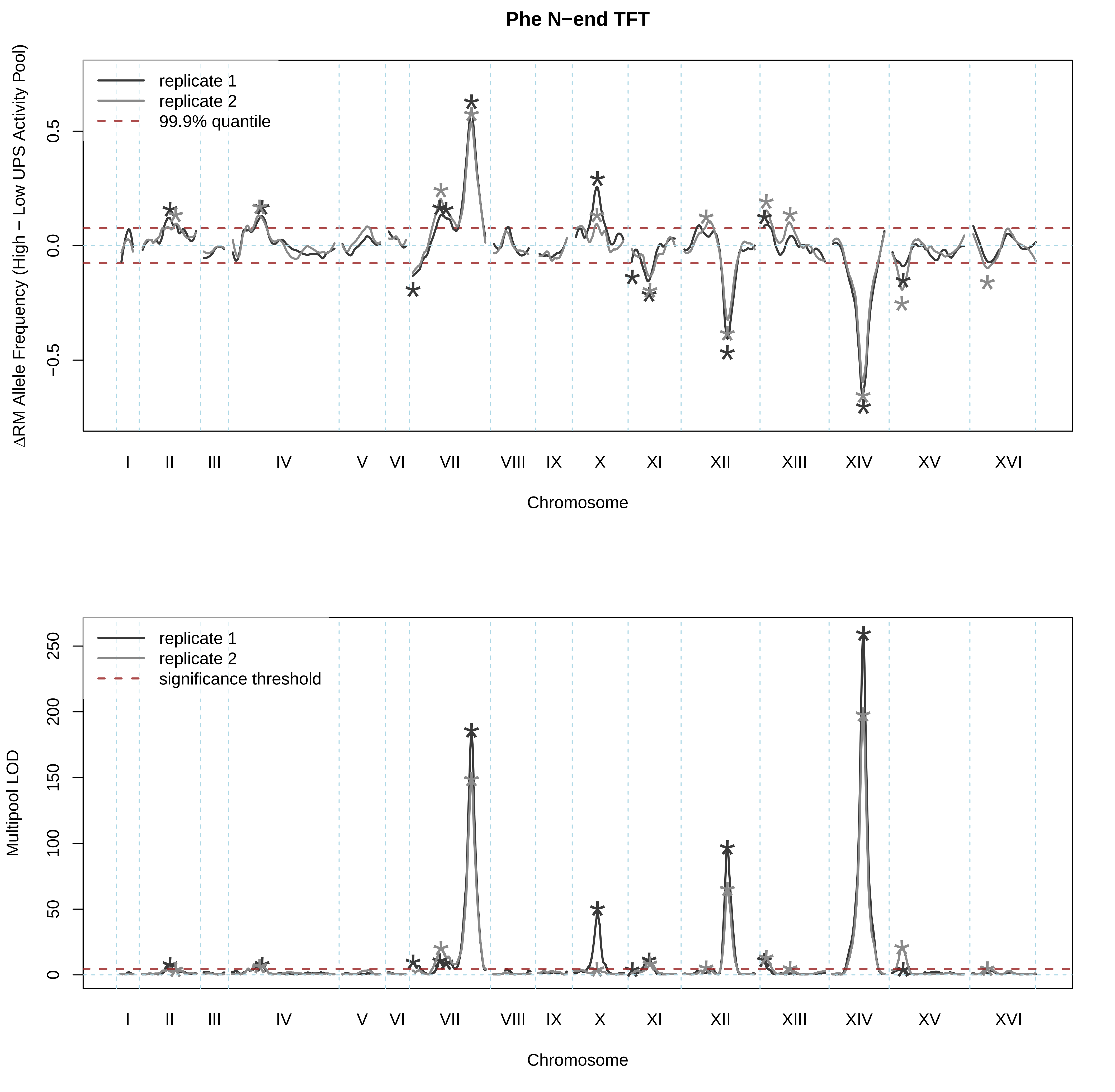

Phe N−end TFT
replicate 1
replicate 2
99.9% quantile
ΔRM Allele Frequency (High − Low UPS Activity Pool)
0.5
0.0
−0.5
I
II
III
IV
V
VI
VII
VIII
IX
X
XI
XII
XIII
XIV
XV
XVI
Chromosome
replicate 1
replicate 2
significance threshold
Multipool LOD
250
200
150
100
50
0
I
II
III
IV
V
VI
VII
VIII
IX
X
XI
XII
XIII
XIV
XV
XVI
Chromosome

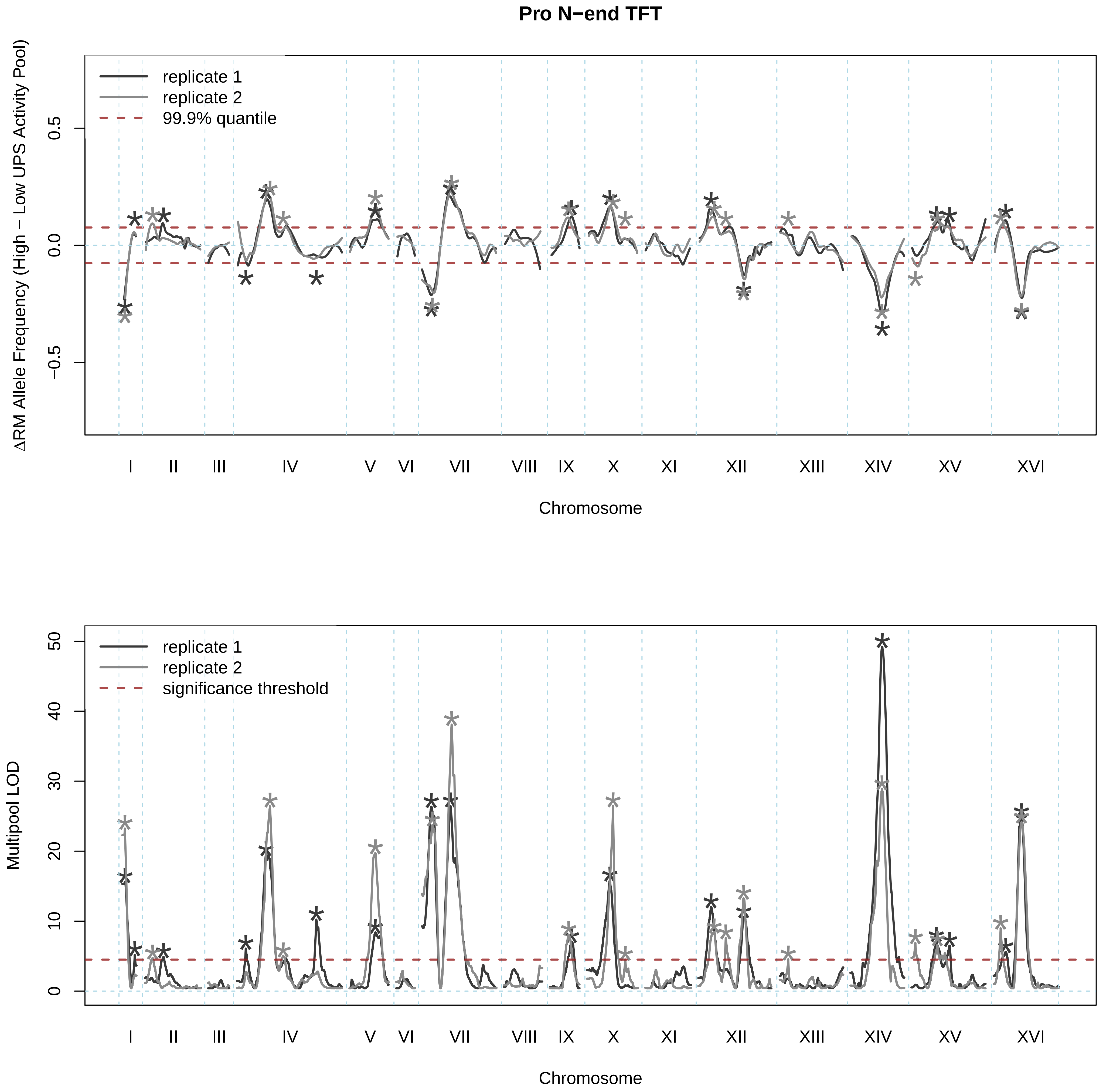

Pro N−end TFT
ΔRM Allele Frequency (High − Low UPS Activity Pool)
replicate 1
replicate 2
99.9% quantile
0.5
0.0
−0.5
I
II
III
IV
V
VI
VII
VIII
IX
X
XI
XII
XIII
XIV
XV
XVI
Chromosome
Multipool LOD
replicate 1
replicate 2
significance threshold
50
40
30
20
10
0
I
II
III
IV
V
VI
VII
VIII
IX
X
XI
XII
XIII
XIV
XV
XVI
Chromosome

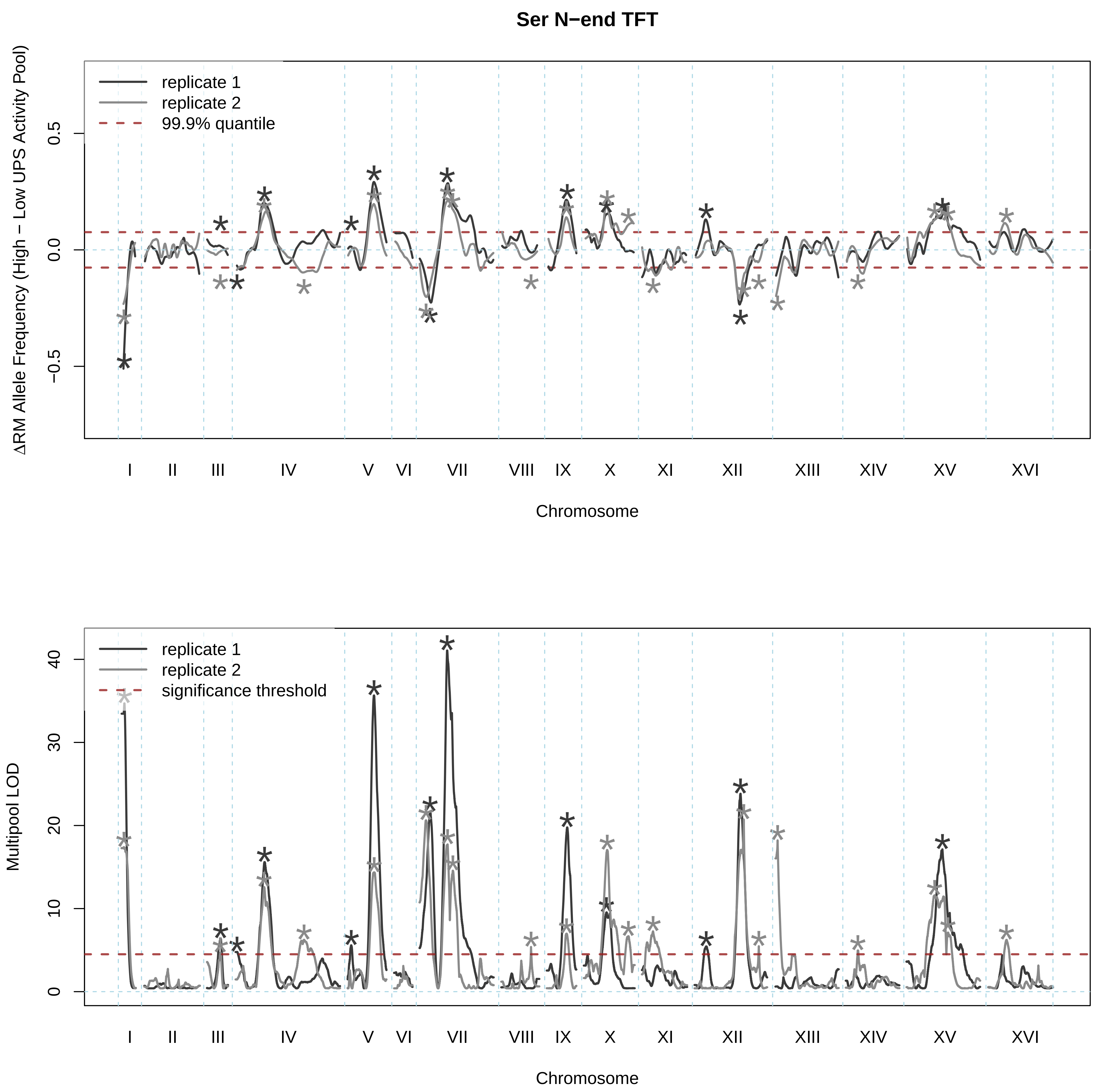

Ser N−end TFT
replicate 1
replicate 2
99.9% quantile
ΔRM Allele Frequency (High − Low UPS Activity Pool)
0.5
0.0
−0.5
I
II
III
IV
V
VI
VII
VIII
IX
X
XI
XII
XIII
XIV
XV
XVI
Chromosome
replicate 1
replicate 2
significance threshold
Multipool LOD
40
30
20
10
0
I
II
III
IV
V
VI
VII
VIII
IX
X
XI
XII
XIII
XIV
XV
XVI
Chromosome

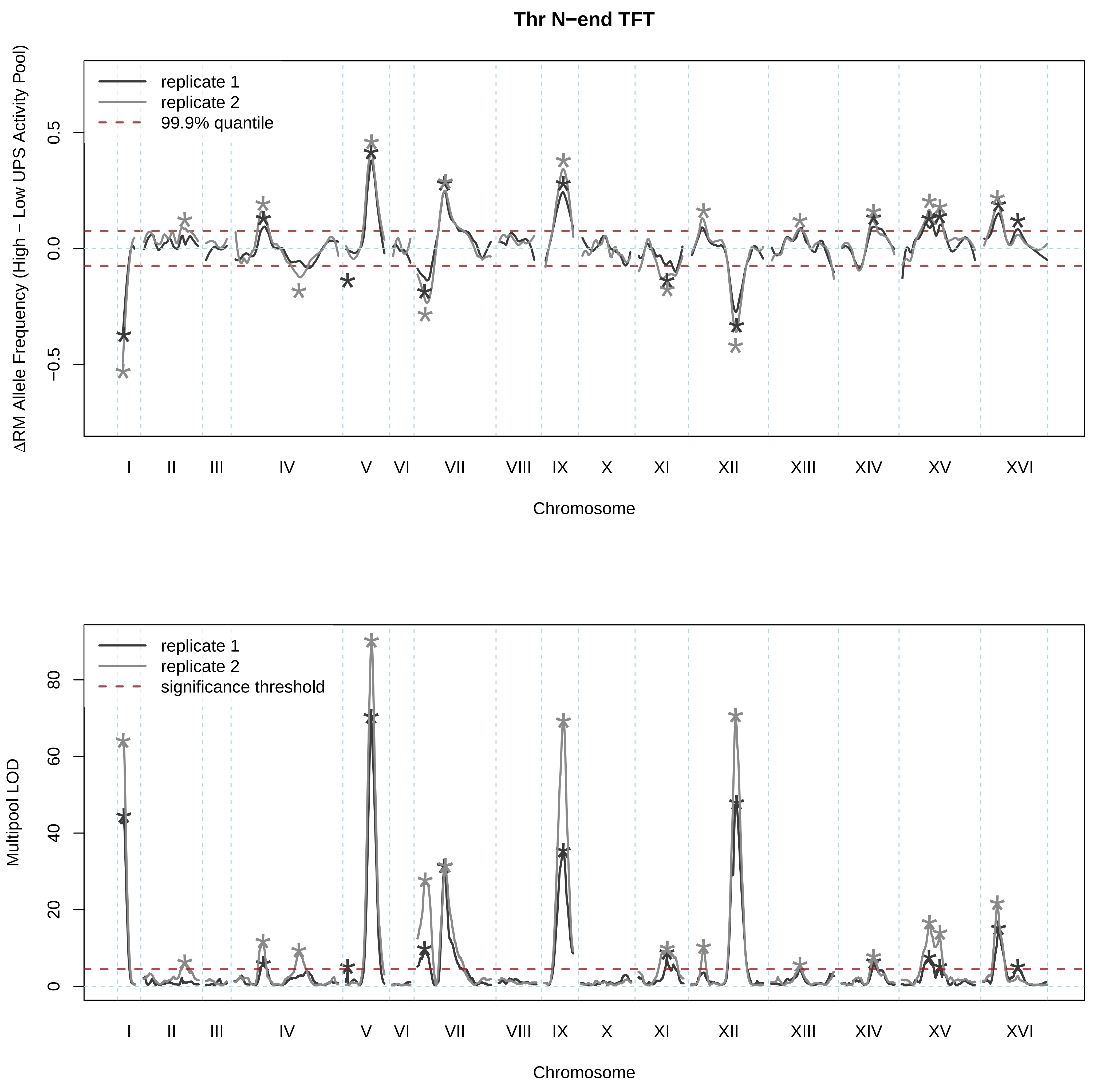

Thr N−end TFT
ΔRM Allele Frequency (High − Low UPS Activity Pool)
replicate 1
replicate 2
99.9% quantile
0.5
0.0
−0.5
I II III IV V VI VII VIII IX X XI XII XIII XIV XV XVI
Chromosome
Multipool LOD
replicate 1
replicate 2
significance threshold
80
60
40
20
0
I II III IV V VI VII VIII IX X XI XII XIII XIV XV XVI
Chromosome

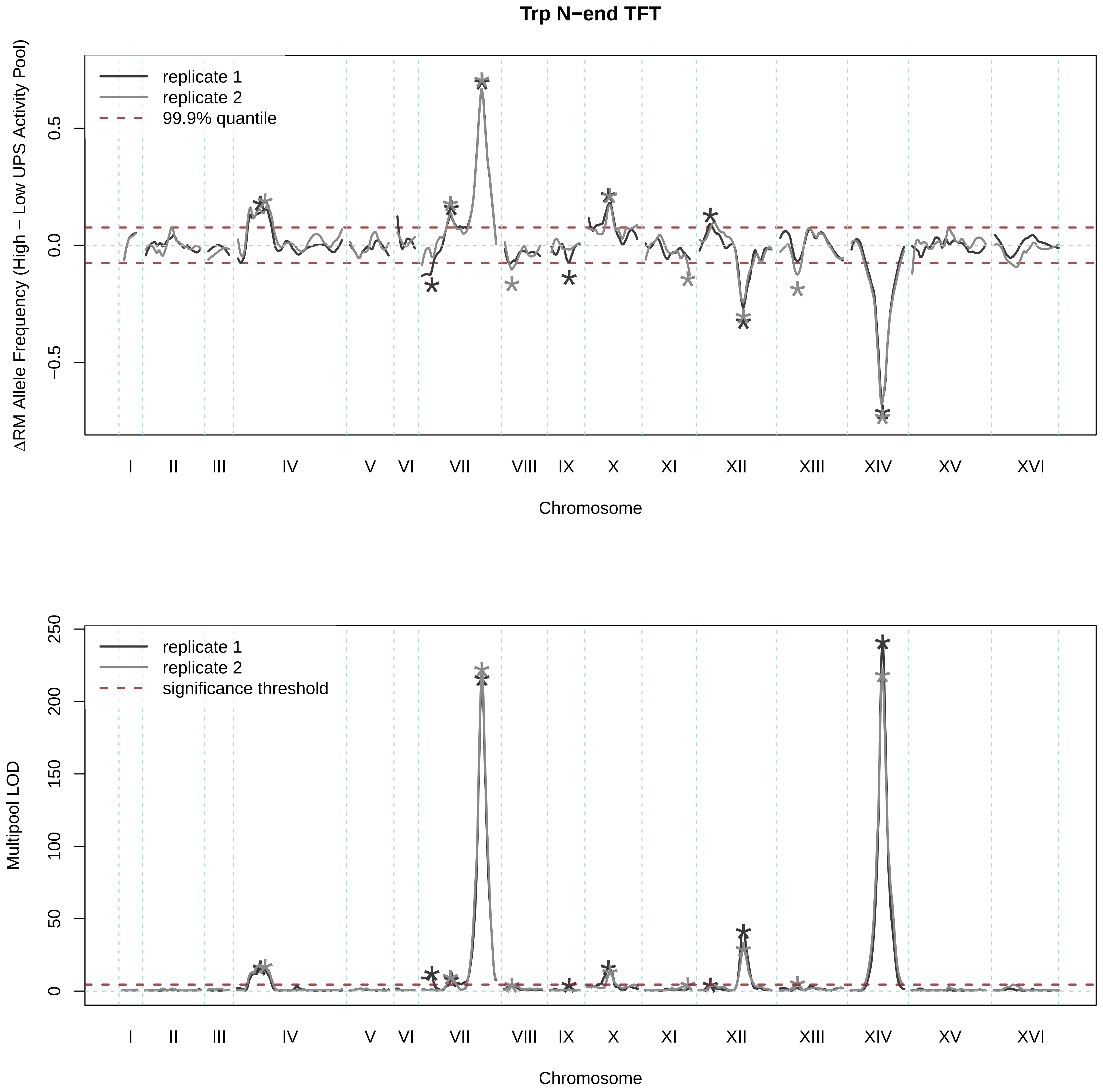

Trp N−end TFT
ΔRM Allele Frequency (High − Low UPS Activity Pool)
replicate 1
replicate 2
99.9% quantile
0.5
0.0
−0.5
I II III IV V VI VII VIII IX X XI XII XIII XIV XV XVI
Chromosome
Multipool LOD
replicate 1
replicate 2
significance threshold
250
200
150
100
50
0
I II III IV V VI VII VIII IX X XI XII XIII XIV XV XVI
Chromosome

Tyr N−end TFT

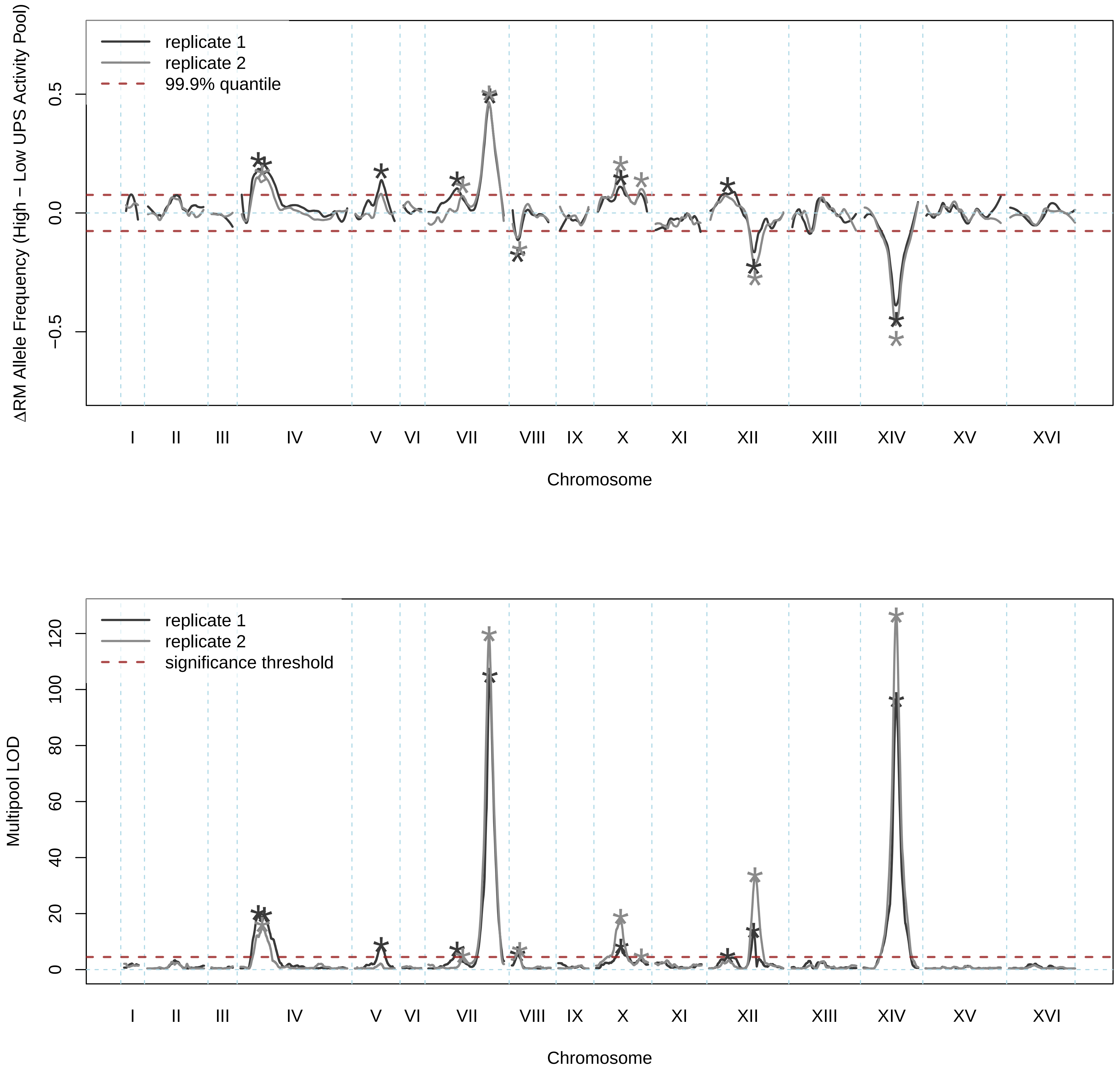

replicate 1
replicate 2
99.9% quantile
ΔRM Allele Frequency (High − Low UPS Activity Pool)
0.5
0.0
−0.5
I
II
III
IV
V
VI
VII
VIII
IX
X
XI
XII
XIII
XIV
XV
XVI
Chromosome
replicate 1
replicate 2
significance threshold
Multipool LOD
0
20
40
60
80
100
120
I
II
III
IV
V
VI
VII
VIII
IX
X
XI
XII
XIII
XIV
XV
XVI
Chromosome

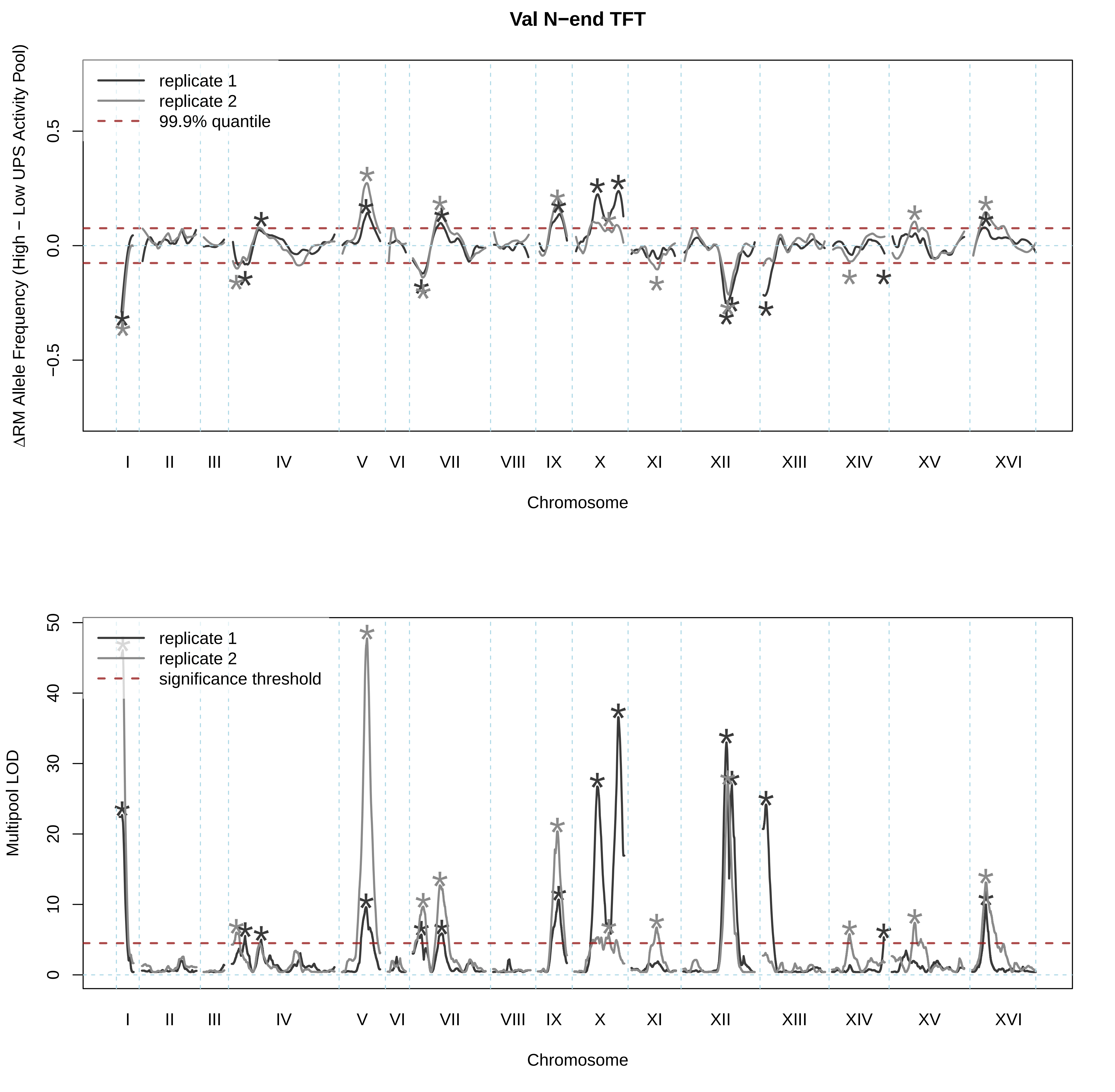

Val N−end TFT
replicate 1
replicate 2
99.9% quantile
ΔRM Allele Frequency (High − Low UPS Activity Pool)
0.5
0.0
−0.5
I
II
III
IV
V
VI
VII
VIII
IX
X
XI
XII
XIII
XIV
XV
XVI
Chromosome
replicate 1
replicate 2
significance threshold
Multipool LOD
50
40
30
20
10
0
I
II
III
IV
V
VI
VII
VIII
IX
X
XI
XII
XIII
XIV
XV
XVI
Chromosome
