# Supplementary material for: Variation in ubiquitin system genes creates substrate-specific effects on proteasomal protein degradation
Source: eLife. 2022 Oct 11;11:e79570. doi: 10.7554/eLife.79570 (PMC9634822; doi:10.7554/eLife.79570)
Supplement: Supplementary file 2. — The LOD score and RM allele frequency difference (QTL effect direction) traces for two independent biological replicates of each N-degron are shown for each of 23 pathway-specific QTL regions. Dashed lines at distinct LOD scores illustrate how changing the significance threshold changes the pathway-specificity of a given QTL region. [file elife-79570-supp2.pdf]

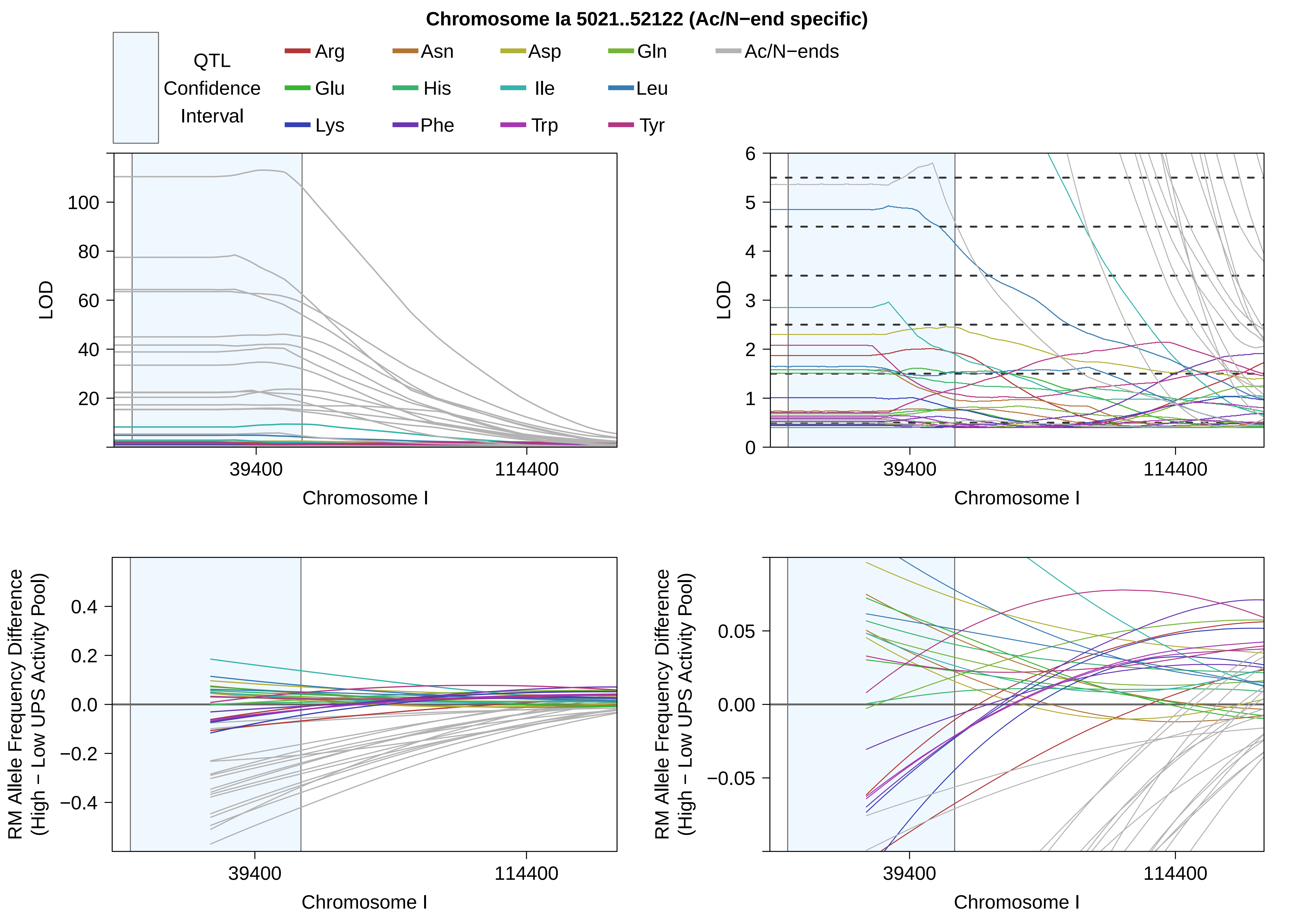

Chromosome Ia 5021..52122 (Ac/N−end specific)
QTL Confidence Interval
Arg
Asn
Asp
Gln
Ac/N−ends
Glu
His
Ile
Leu
Lys
Phe
Trp
Tyr
LOD
Chromosome I
39400
114400
RM Allele Frequency Difference (High – Low UPS Activity Pool)

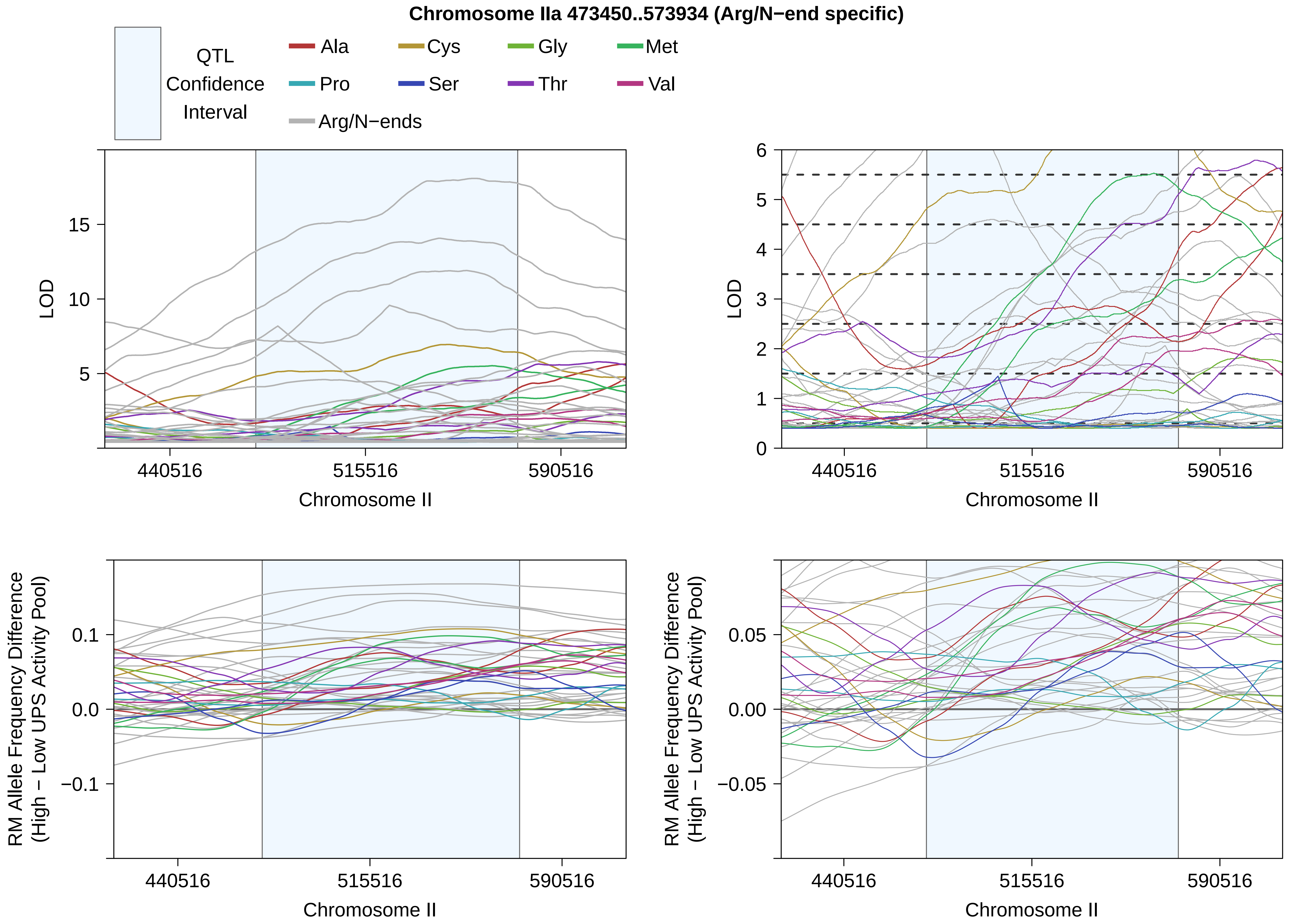
Chromosome IIa 473450..573934 (Arg/N−end specific)
QTL Confidence Interval
Ala
Cys
Gly
Met
Pro
Ser
Thr
Val
Arg/N−ends
LOD
Chromosome II
440516
515516
590516
RM Allele Frequency Difference (High − Low UPS Activity Pool)

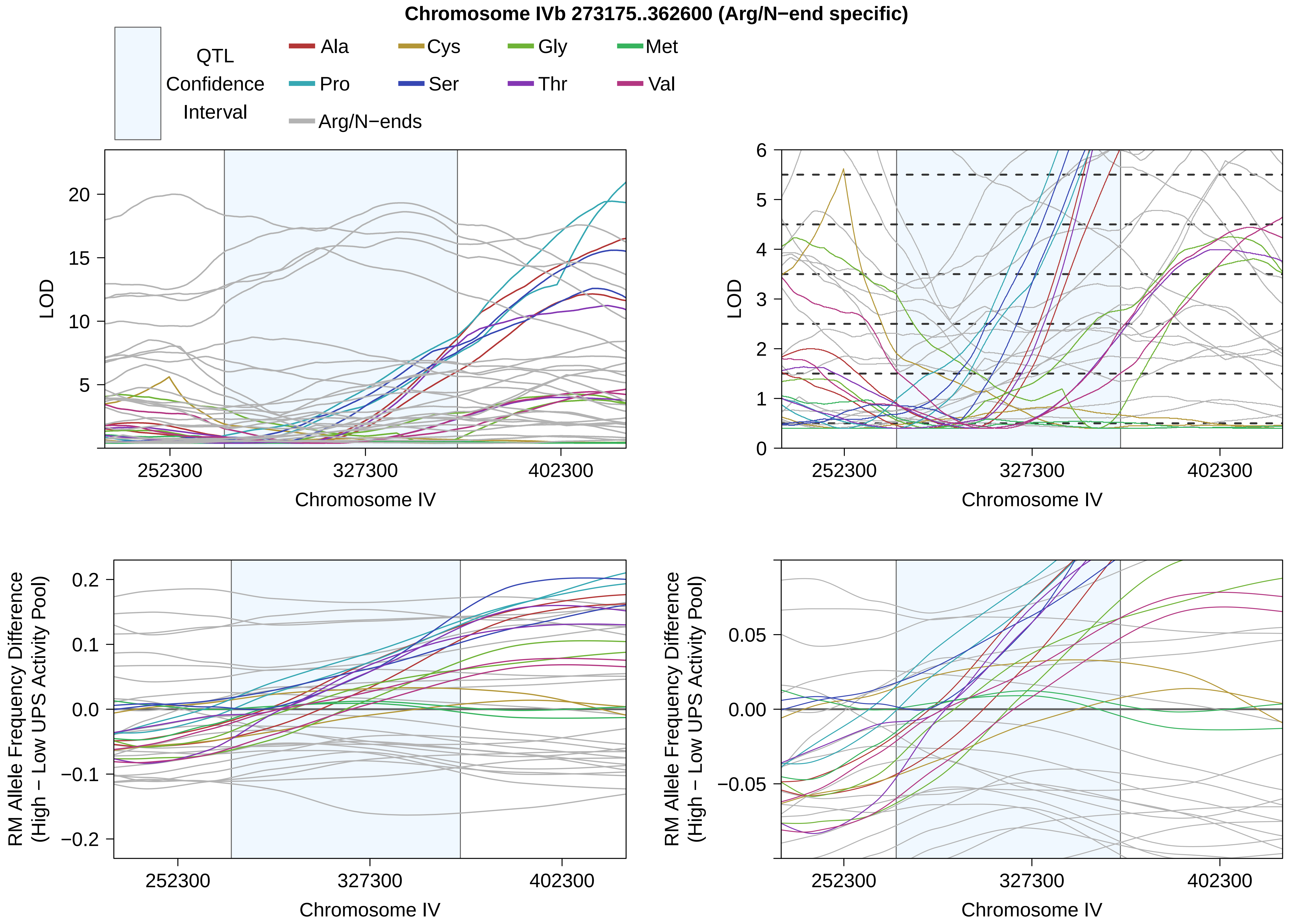

Chromosome IVb 273175..362600 (Arg/N−end specific)
QTL Confidence Interval
Ala
Cys
Gly
Met
Pro
Ser
Thr
Val
Arg/N−ends
LOD
Chromosome IV
252300
327300
402300
RM Allele Frequency Difference (High − Low UPS Activity Pool)

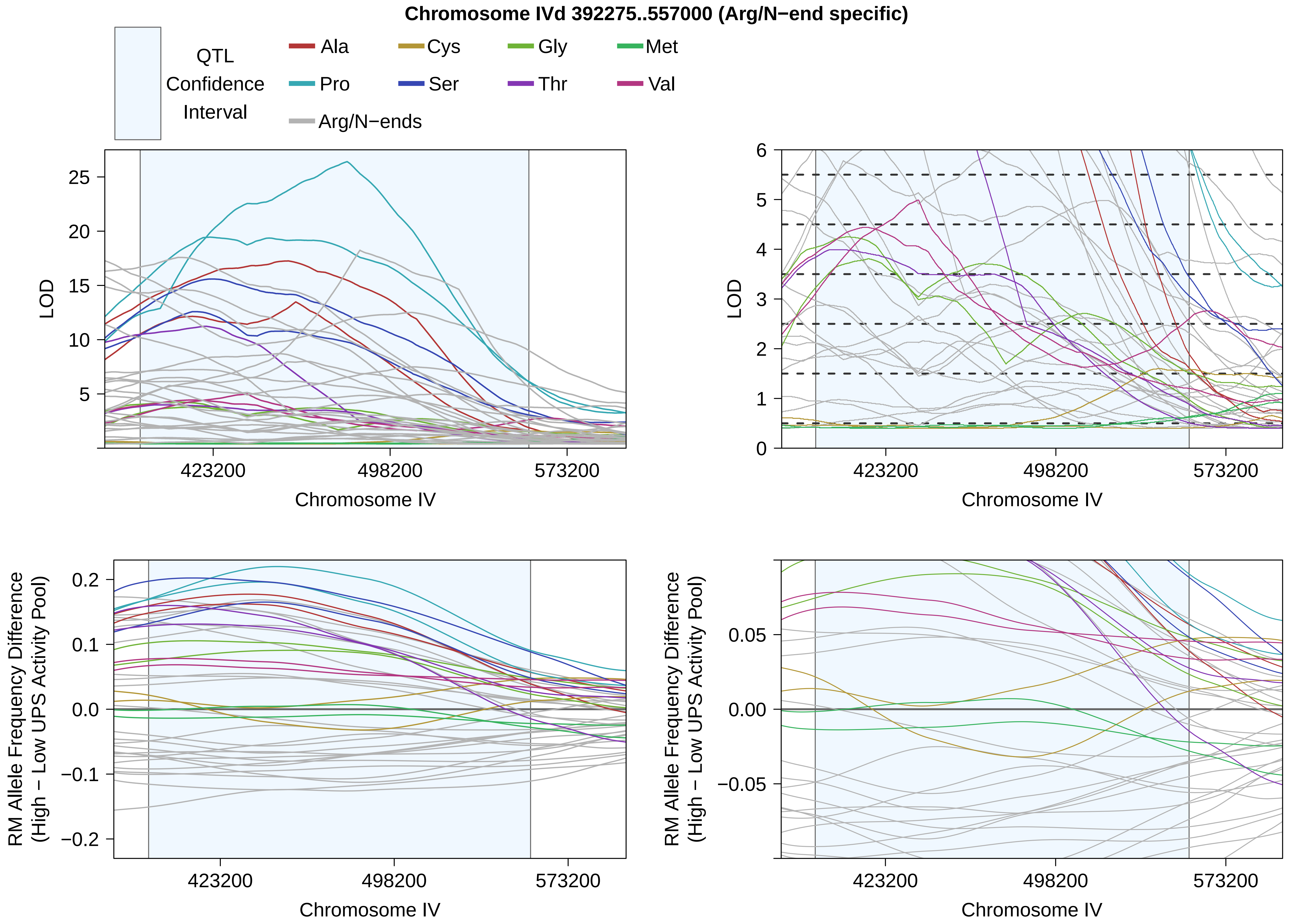

Chromosome IVd 392275..557000 (Arg/N−end specific)
QTL Confidence Interval
Ala
Cys
Gly
Met
Pro
Ser
Thr
Val
Arg/N−ends
LOD
25
20
15
10
5
6
4
3
2
1
0
423200
498200
573200
Chromosome IV
RM Allele Frequency Difference (High − Low UPS Activity Pool)
0.2
0.1
0.0
−0.1
−0.2
0.05
0.00
−0.05

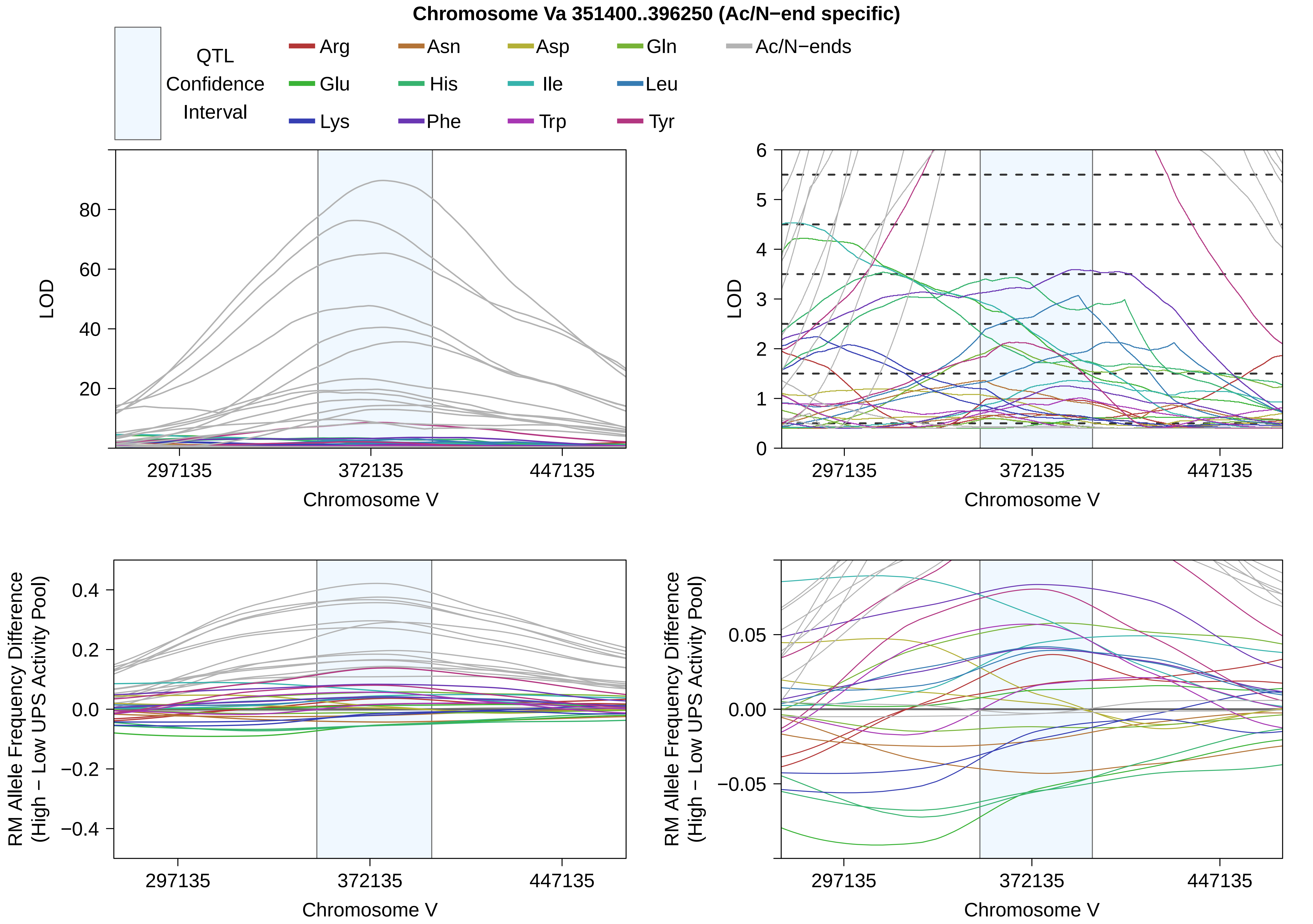

Chromosome Va 351400..396250 (Ac/N−end specific)
QTL
Confidence
Interval
Arg
Asn
Asp
Gln
Ac/N−ends
Glu
His
Ile
Leu
Lys
Phe
Trp
Tyr
LOD
Chromosome V
297135
372135
447135
RM Allele Frequency Difference
(High − Low UPS Activity Pool)

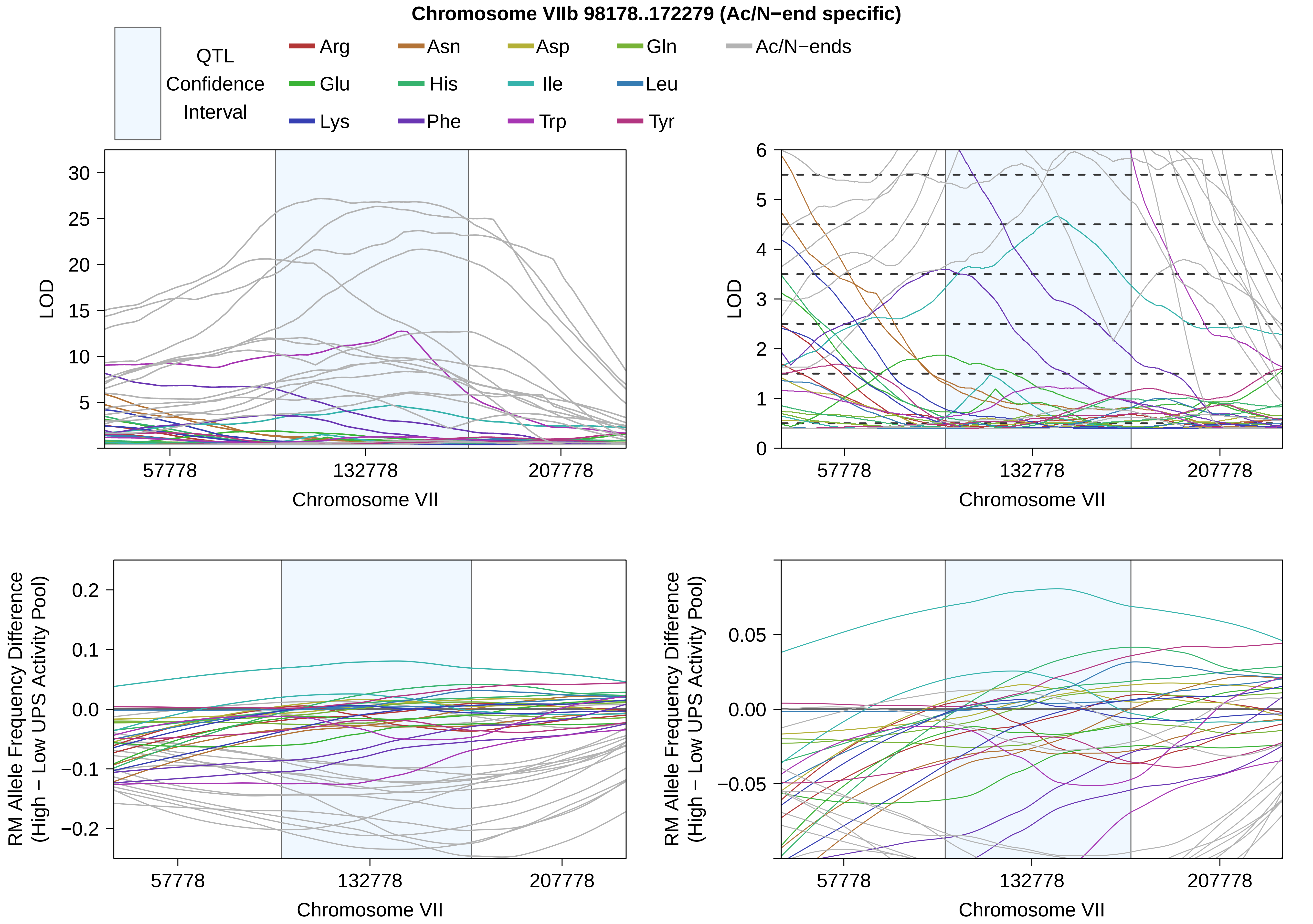

Chromosome VIIb 98178..172279 (Ac/N−end specific)
QTL Confidence Interval
Arg
Asn
Asp
Gln
Ac/N−ends
Glu
His
Ile
Leu
Lys
Phe
Trp
Tyr
LOD
Chromosome VII
57778
132778
207778
RM Allele Frequency Difference (High − Low UPS Activity Pool)

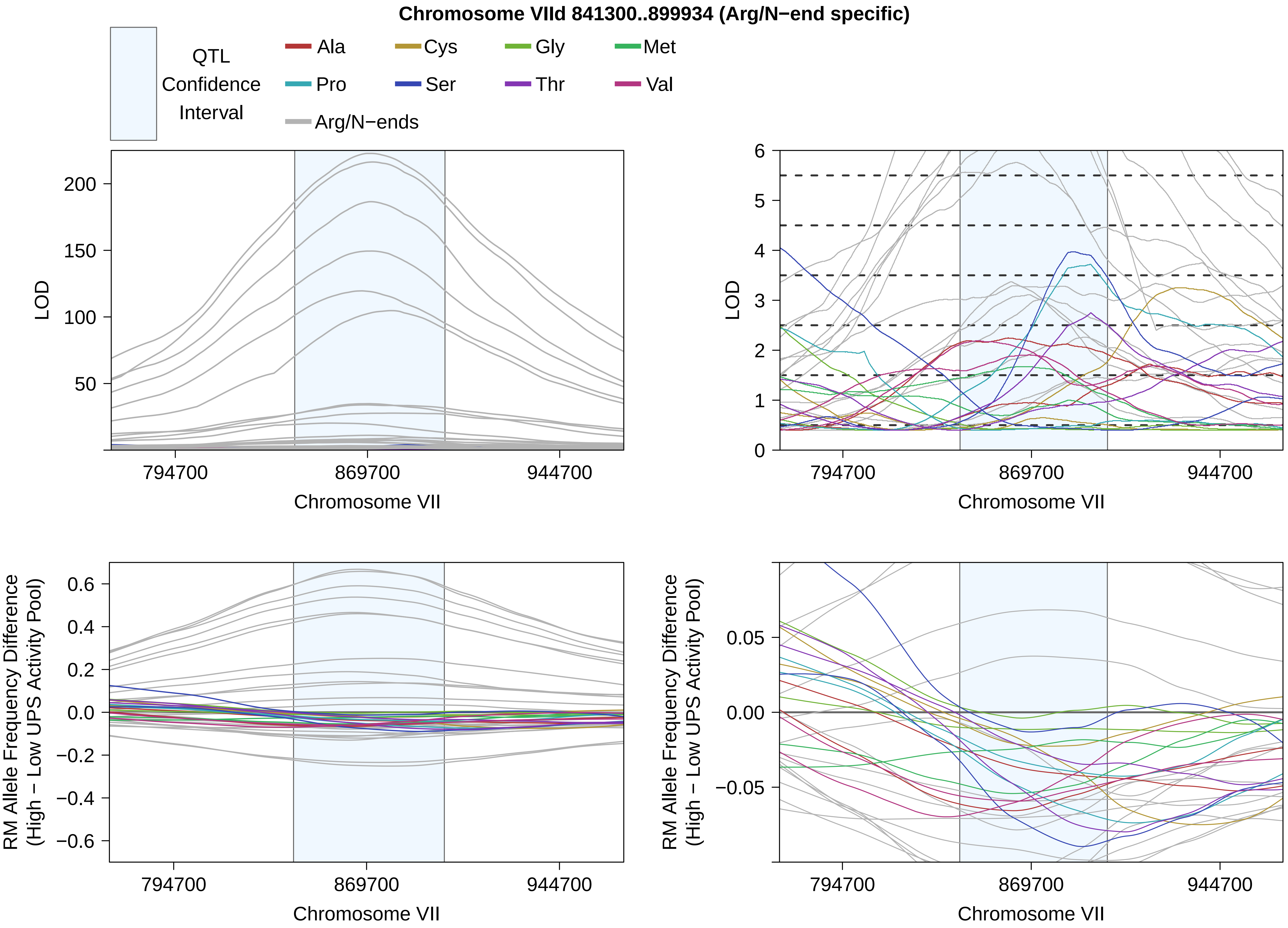

Chromosome VIId 841300..899934 (Arg/N−end specific)
QTL Confidence Interval
Ala
Cys
Gly
Met
Pro
Ser
Thr
Val
Arg/N−ends
LOD
200
150
100
50
0
1
2
3
4
5
6
794700
869700
944700
Chromosome VII
RM Allele Frequency Difference (High − Low UPS Activity Pool)
0.6
0.4
0.2
0.0
−0.2
−0.4
−0.6
0.05
0.00
−0.05

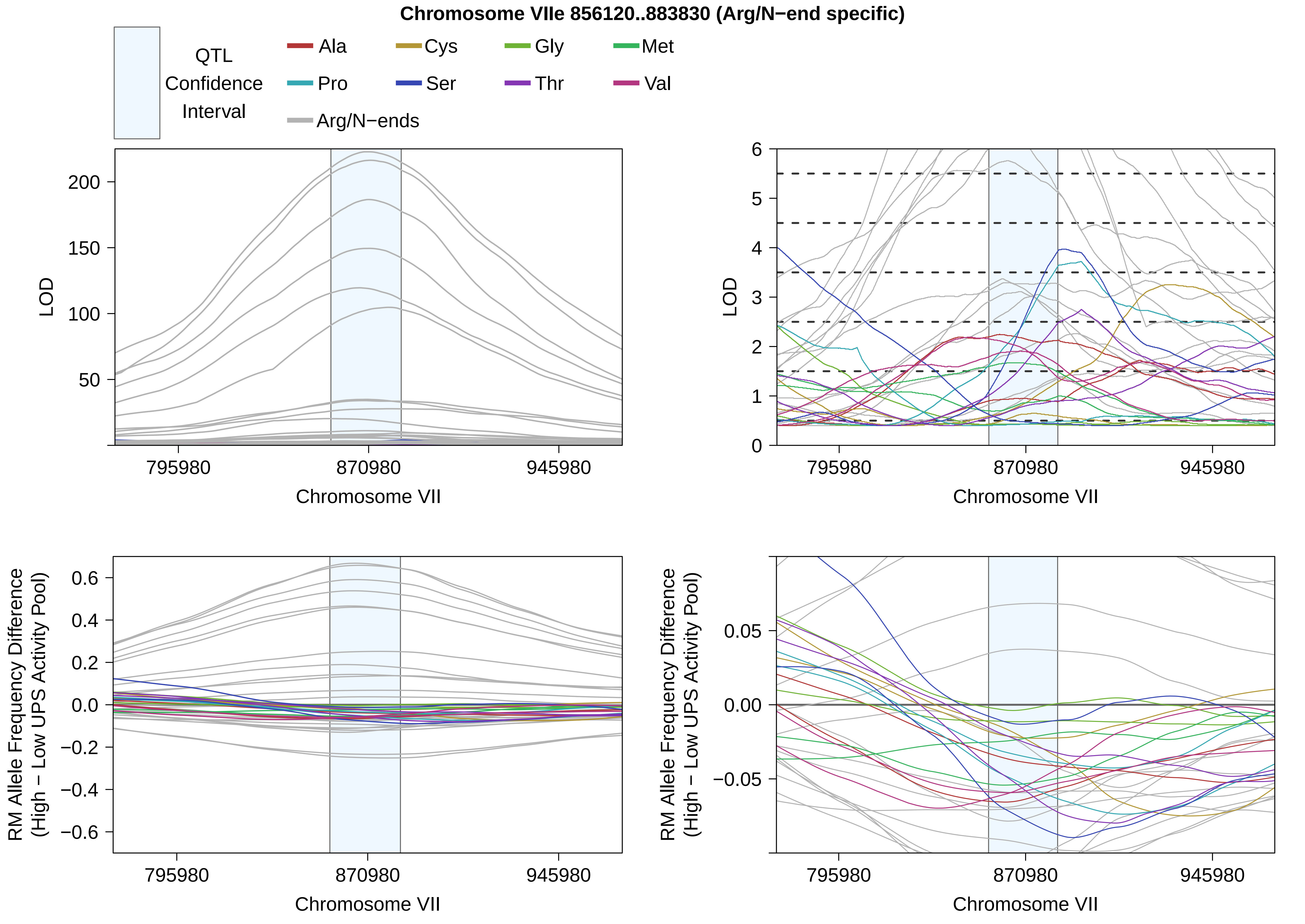

Chromosome VIIe 856120..883830 (Arg/N−end specific)
QTL Confidence Interval
Ala
Cys
Gly
Met
Pro
Ser
Thr
Val
Arg/N−ends
LOD
Chromosome VII
795980
870980
945980
RM Allele Frequency Difference (High − Low UPS Activity Pool)

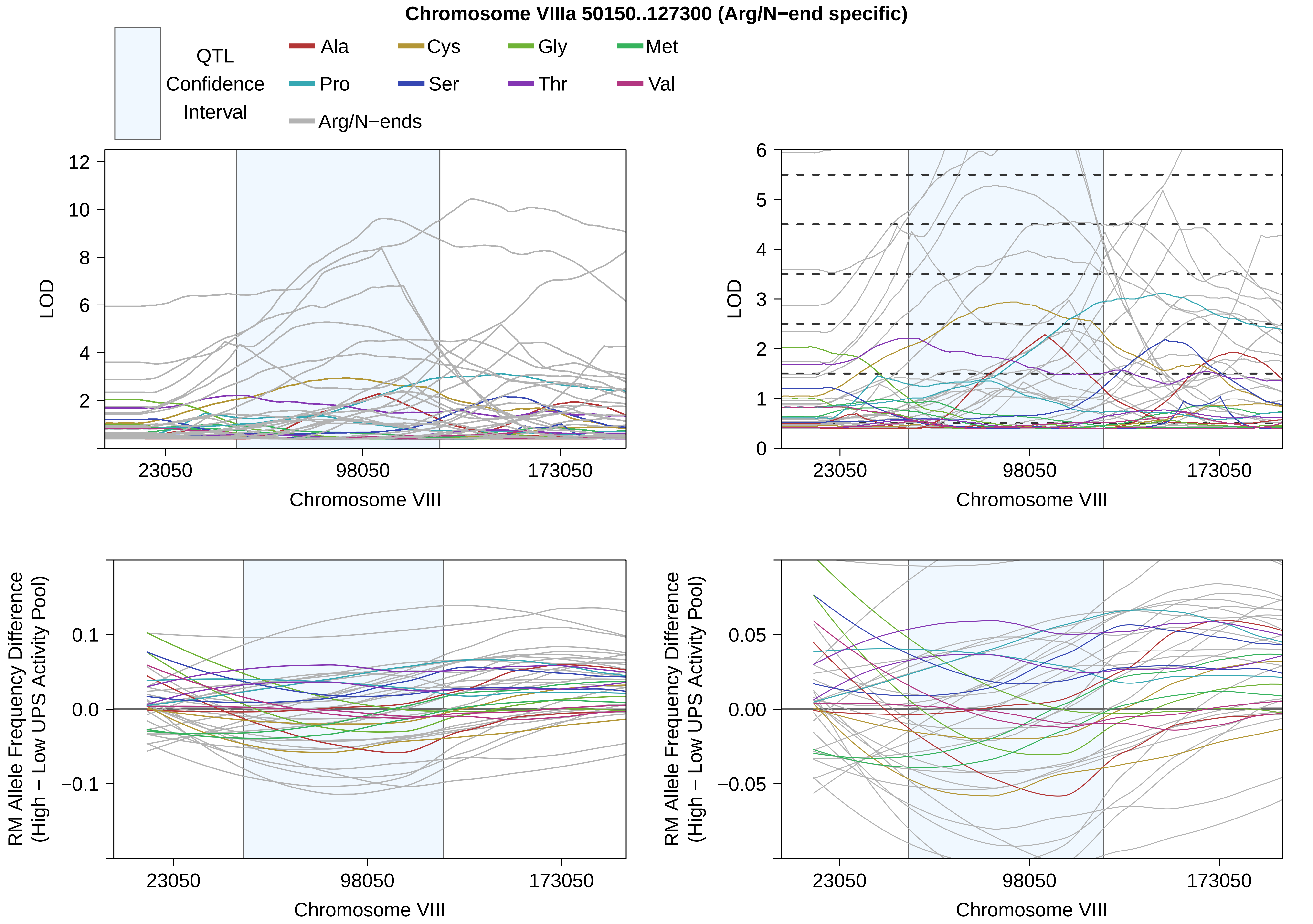

Chromosome VIIIa 50150..127300 (Arg/N−end specific)
QTL
Confidence
Interval
Ala
Cys
Gly
Met
Pro
Ser
Thr
Val
Arg/N−ends
LOD
12
10
8
6
4
2
0
1
3
5
23050
98050
173050
Chromosome VIII
RM Allele Frequency Difference
(High − Low UPS Activity Pool)
0.1
0.0
−0.1
0.05
0.00
−0.05

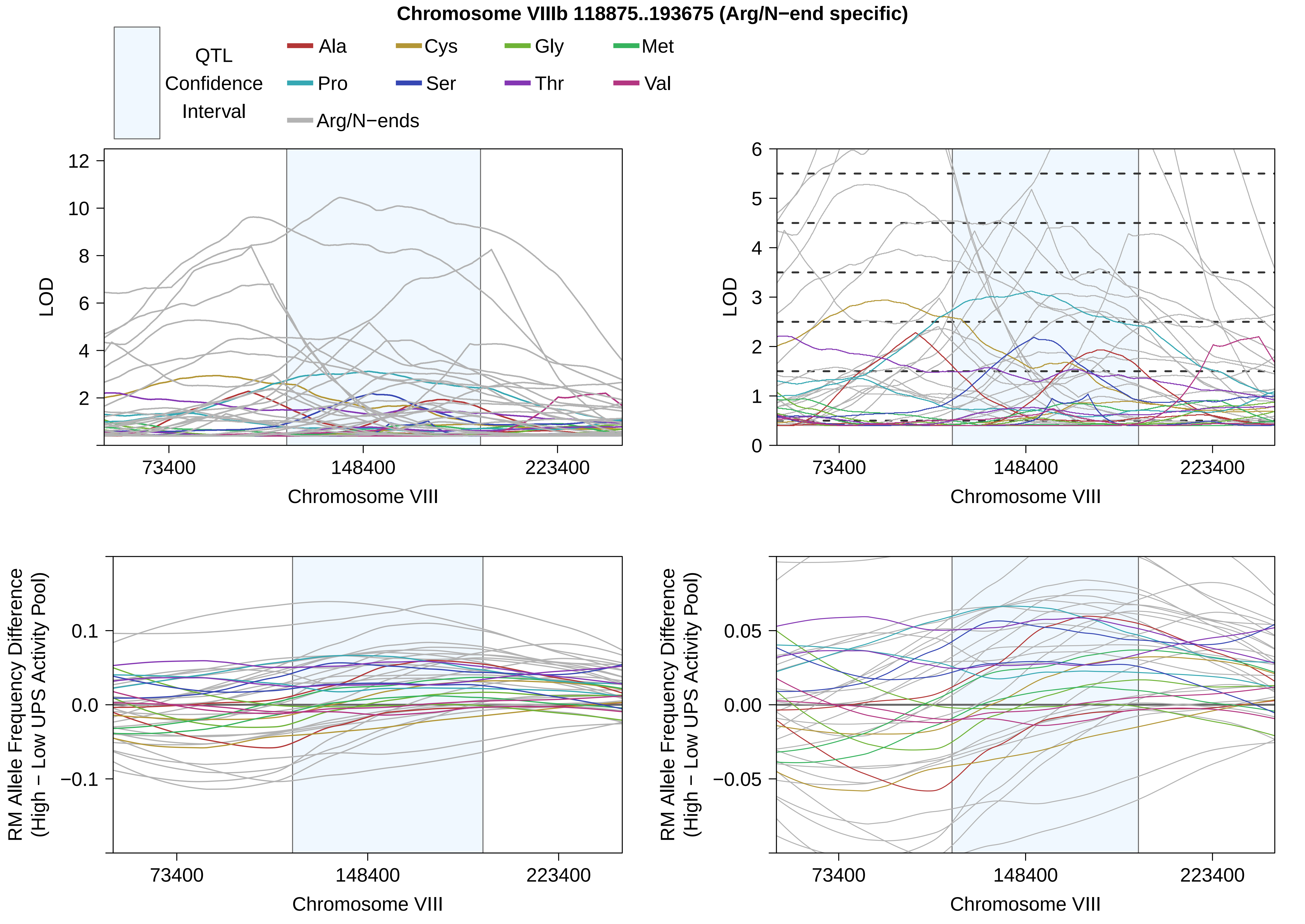

Chromosome VIIIb 118875..193675 (Arg/N−end specific)
QTL Confidence Interval
Ala
Cys
Gly
Met
Pro
Ser
Thr
Val
Arg/N−ends
LOD
Chromosome VIII
73400
148400
223400
RM Allele Frequency Difference (High − Low UPS Activity Pool)

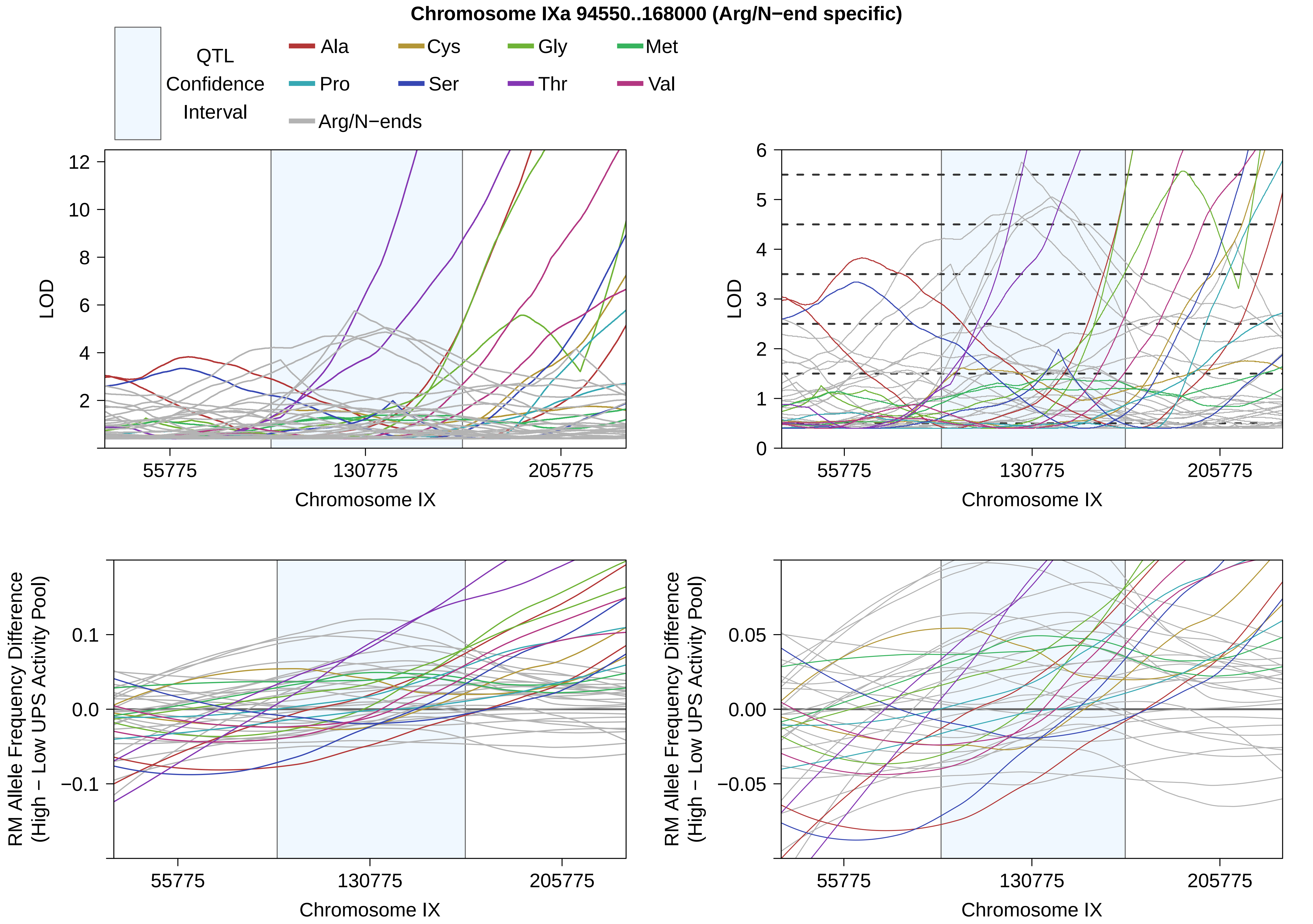

Chromosome IXa 94550..168000 (Arg/N−end specific)
QTL Confidence Interval
Ala
Cys
Gly
Met
Pro
Ser
Thr
Val
Arg/N−ends
LOD
Chromosome IX
RM Allele Frequency Difference (High – Low UPS Activity Pool)
55775
130775
205775

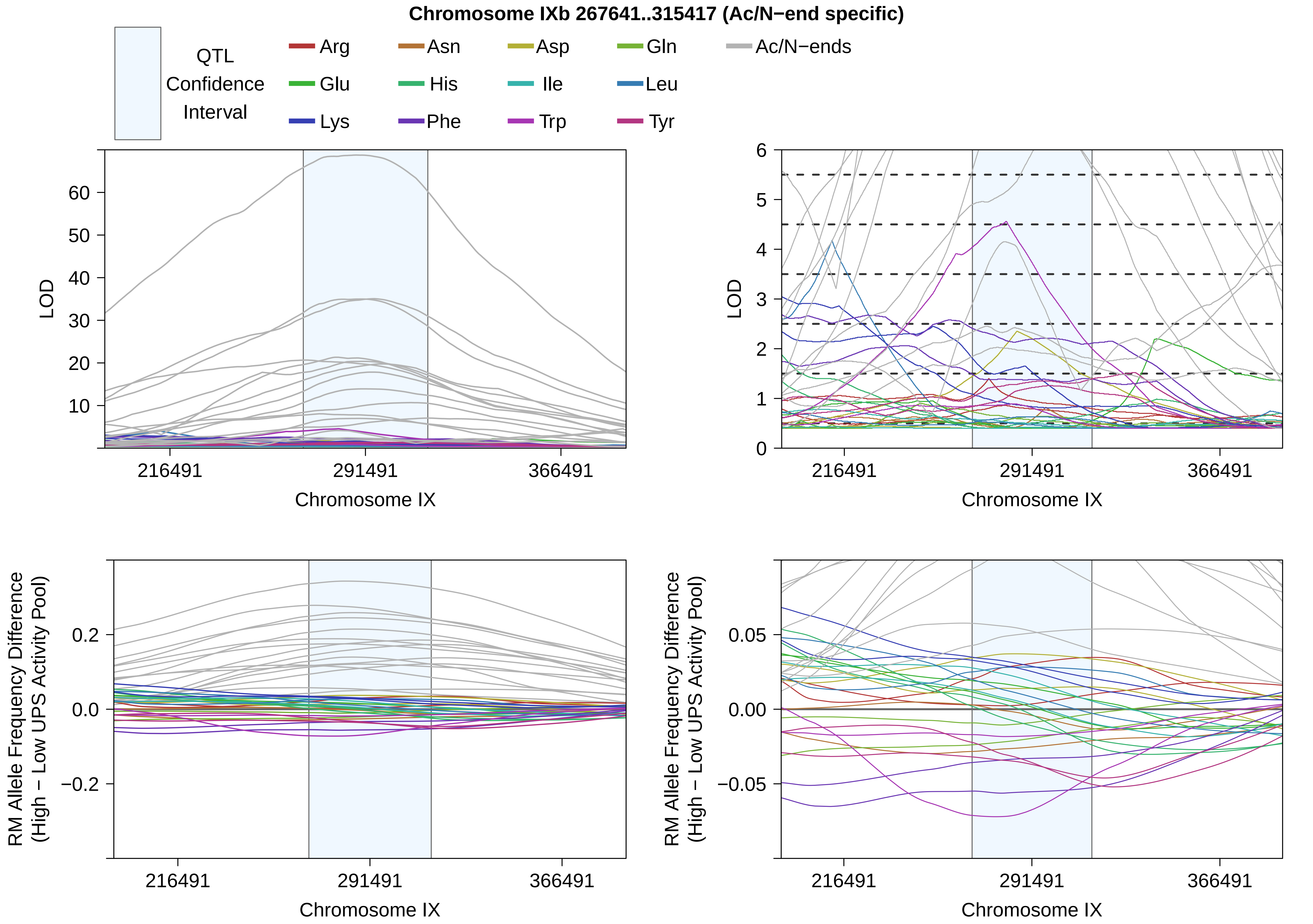

Chromosome IXb 267641..315417 (Ac/N−end specific)
QTL Confidence Interval
Arg
Asn
Asp
Gln
Ac/N−ends
Glu
His
Ile
Leu
Lys
Phe
Trp
Tyr
LOD
Chromosome IX
216491
291491
366491
RM Allele Frequency Difference (High − Low UPS Activity Pool)

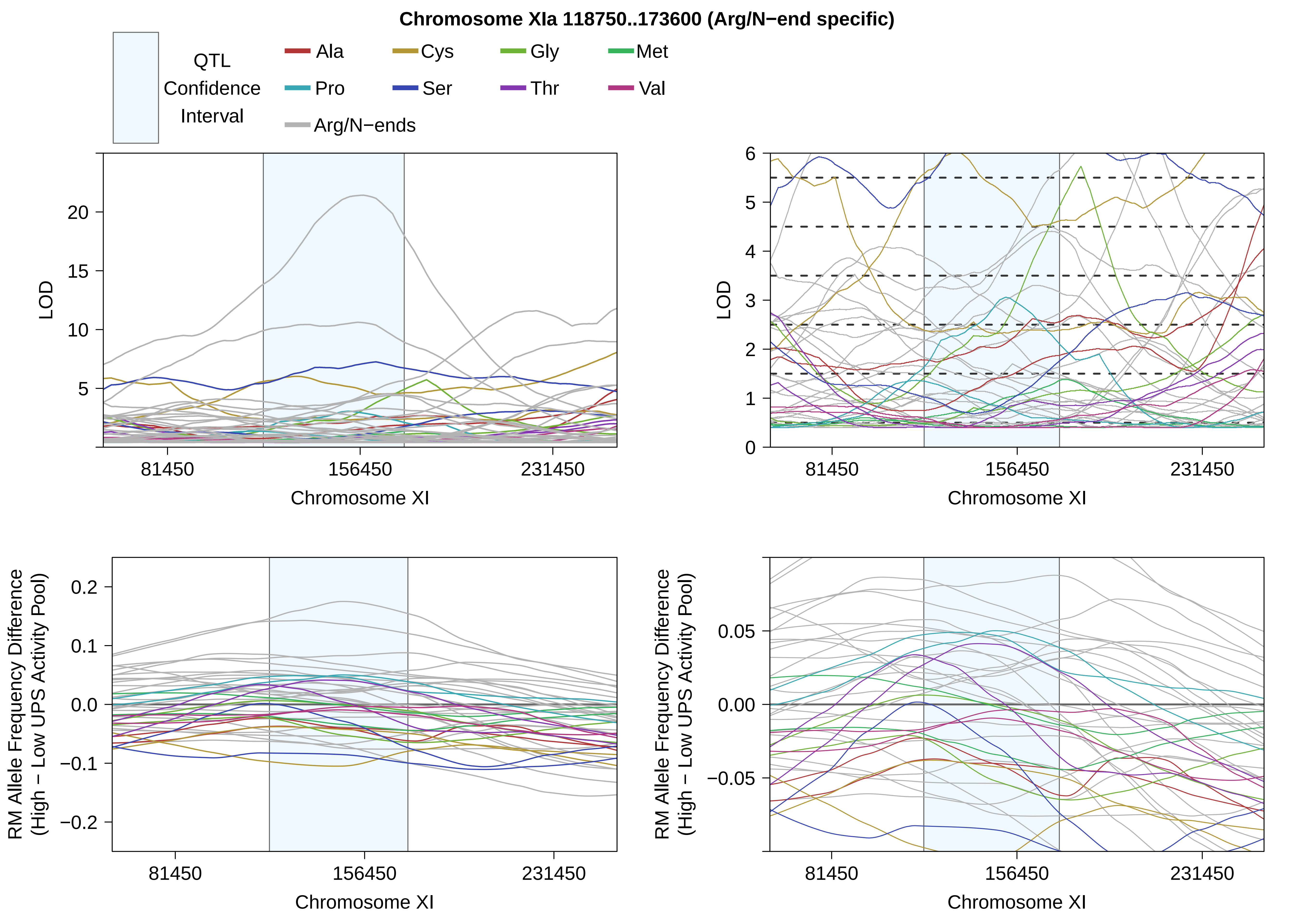

Chromosome XIa 118750..173600 (Arg/N−end specific)
QTL Confidence Interval
Ala
Cys
Gly
Met
Pro
Ser
Thr
Val
Arg/N−ends
LOD
Chromosome XI
81450
156450
231450
RM Allele Frequency Difference (High – Low UPS Activity Pool)

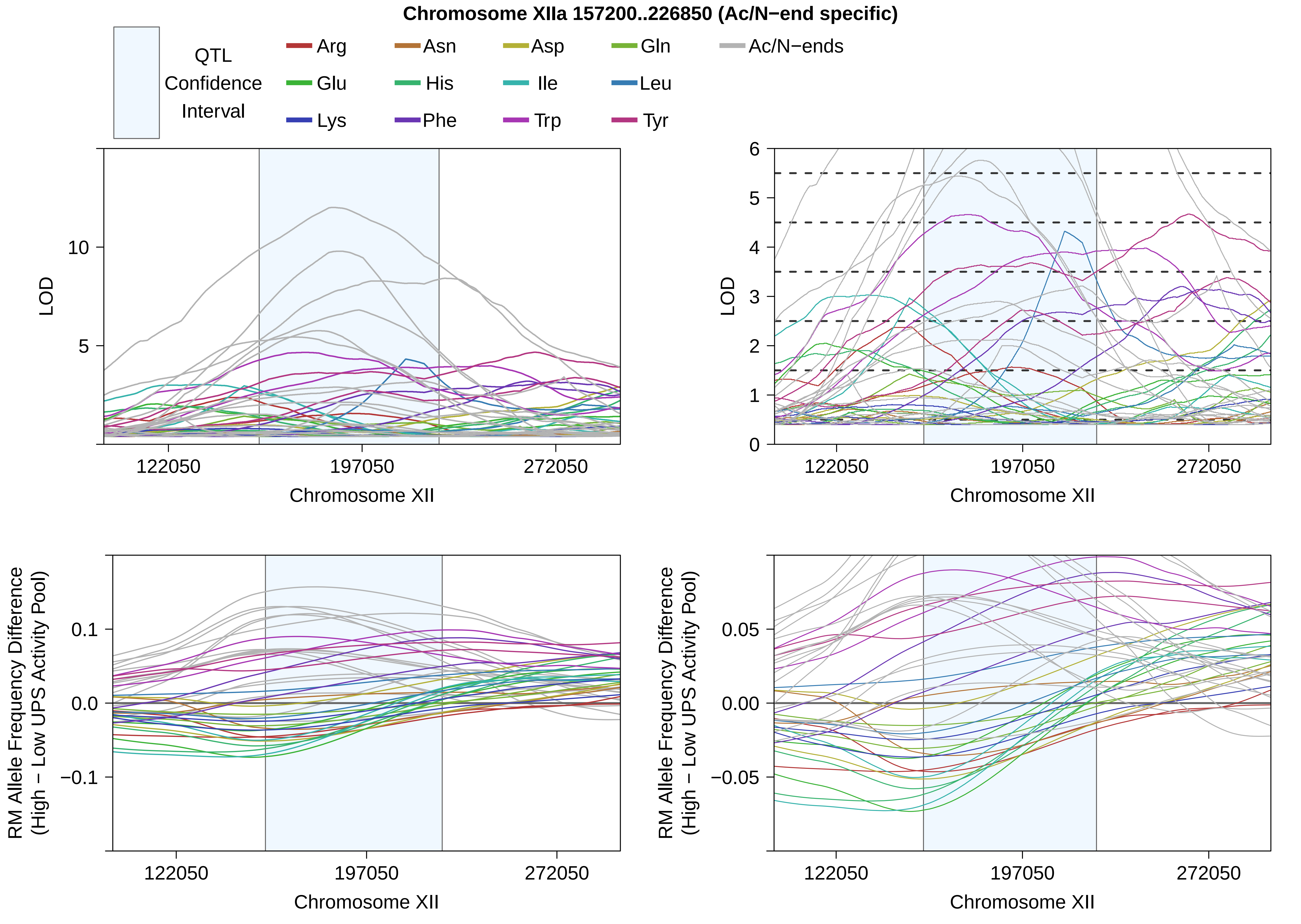

Chromosome XIIa 157200..226850 (Ac/N−end specific)
QTL Confidence Interval
Arg
Asn
Asp
Gln
Ac/N−ends
Glu
His
Ile
Leu
Lys
Phe
Trp
Tyr
LOD
Chromosome XII
122050
197050
272050
RM Allele Frequency Difference (High − Low UPS Activity Pool)

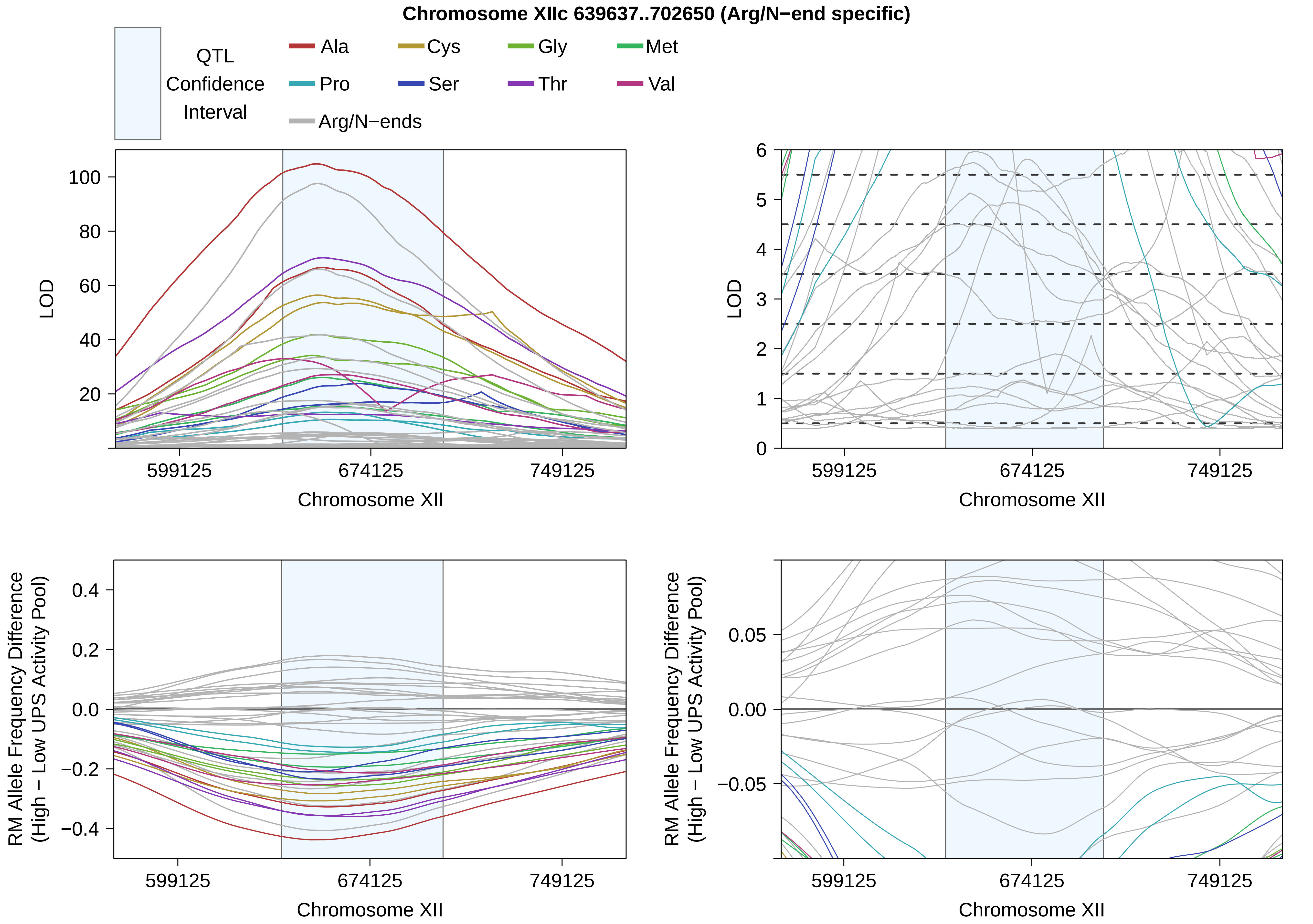
Chromosome XIIc 639637..702650 (Arg/N−end specific)
QTL
Confidence
Interval
Ala
Cys
Gly
Met
Pro
Ser
Thr
Val
Arg/N−ends
LOD
Chromosome XII
599125
674125
749125
RM Allele Frequency Difference
(High − Low UPS Activity Pool)

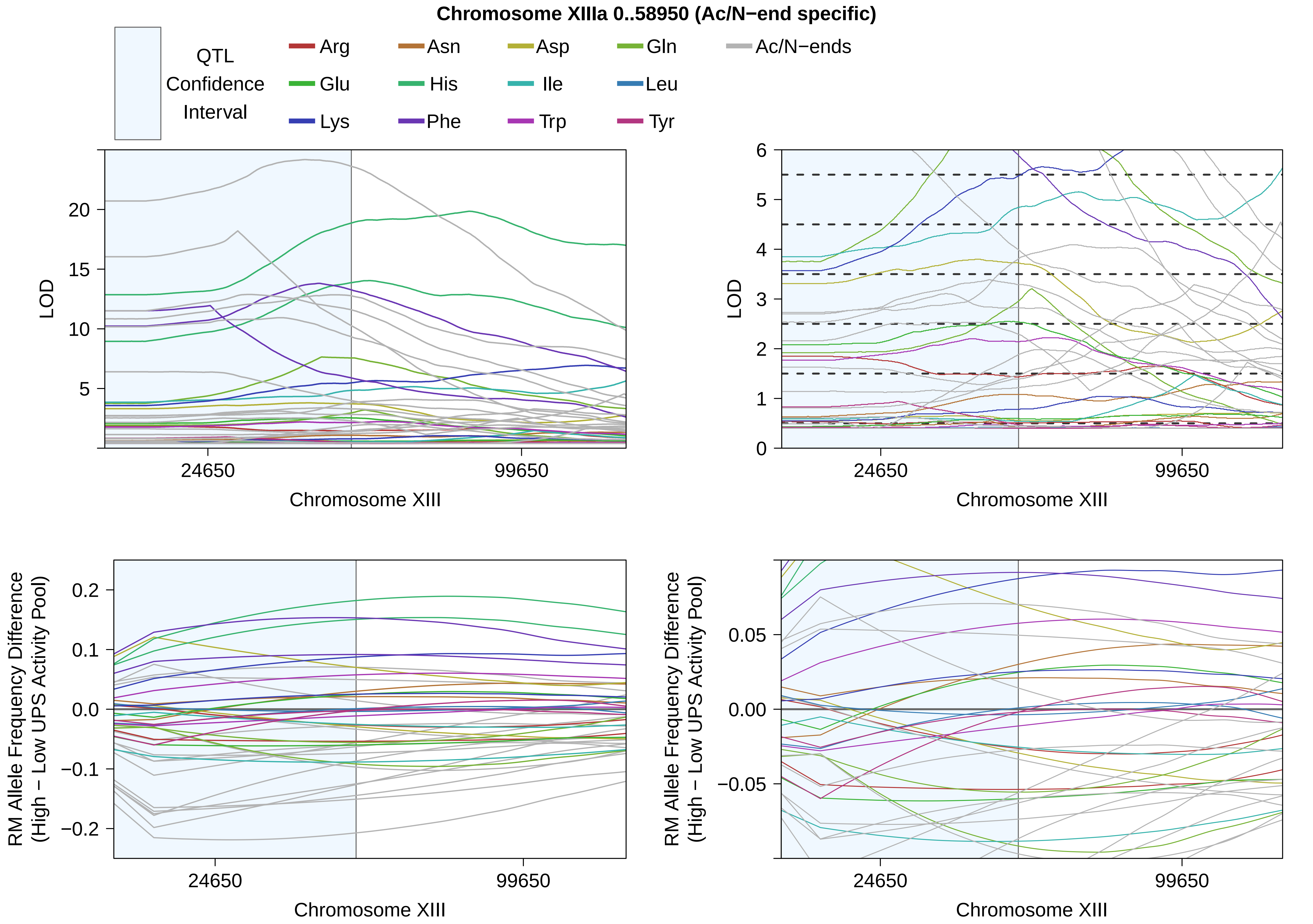

Chromosome XIIIa 0..58950 (Ac/N−end specific)
QTL
Confidence
Interval
Arg
Asn
Asp
Gln
Ac/N−ends
Glu
His
Ile
Leu
Lys
Phe
Trp
Tyr
LOD
20
15
10
5
24650
99650
Chromosome XIII
6
5
4
3
2
1
0
RM Allele Frequency Difference
(High − Low UPS Activity Pool)
0.2
0.1
0.0
−0.1
−0.2
0.05
0.00
−0.05

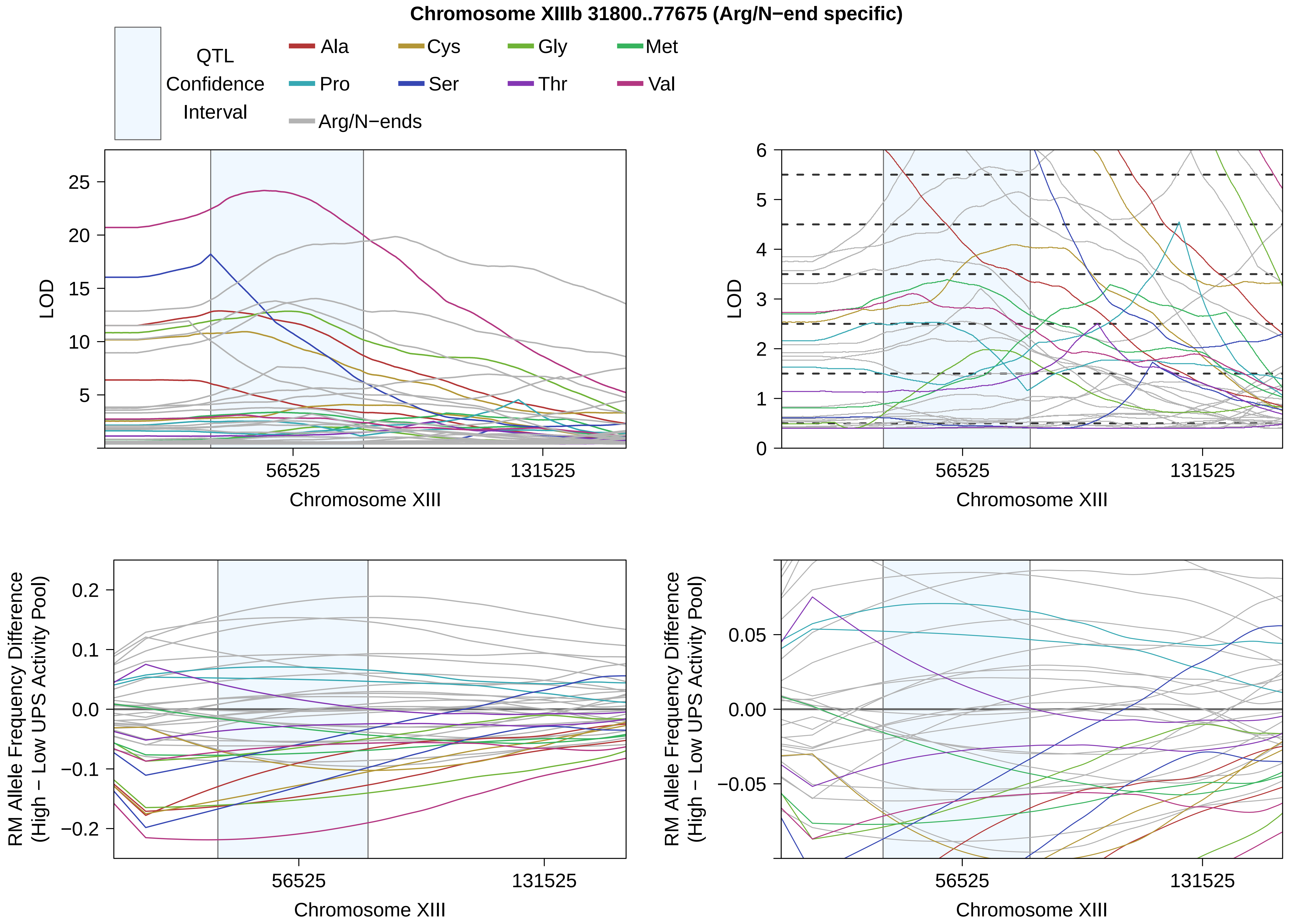

Chromosome XIIIb 31800..77675 (Arg/N−end specific)
QTL Confidence Interval
Ala
Cys
Gly
Met
Pro
Ser
Thr
Val
Arg/N−ends
LOD
Chromosome XIII
56525
131525
RM Allele Frequency Difference (High − Low UPS Activity Pool)

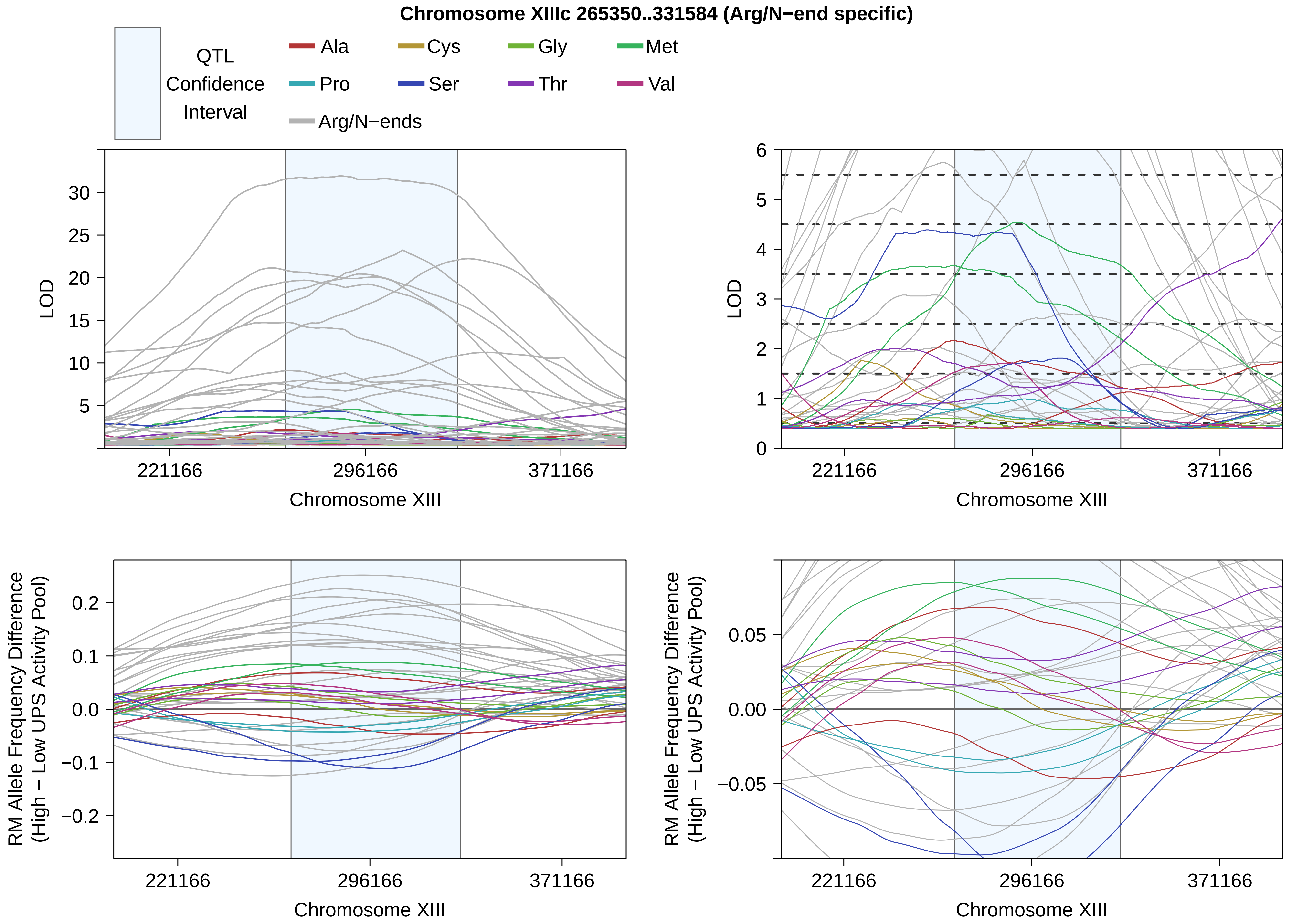

Chromosome XIIIc 265350..331584 (Arg/N−end specific)
QTL Confidence Interval
Ala
Cys
Gly
Met
Pro
Ser
Thr
Val
Arg/N−ends
LOD
Chromosome XIII
221166
296166
371166
RM Allele Frequency Difference (High – Low UPS Activity Pool)

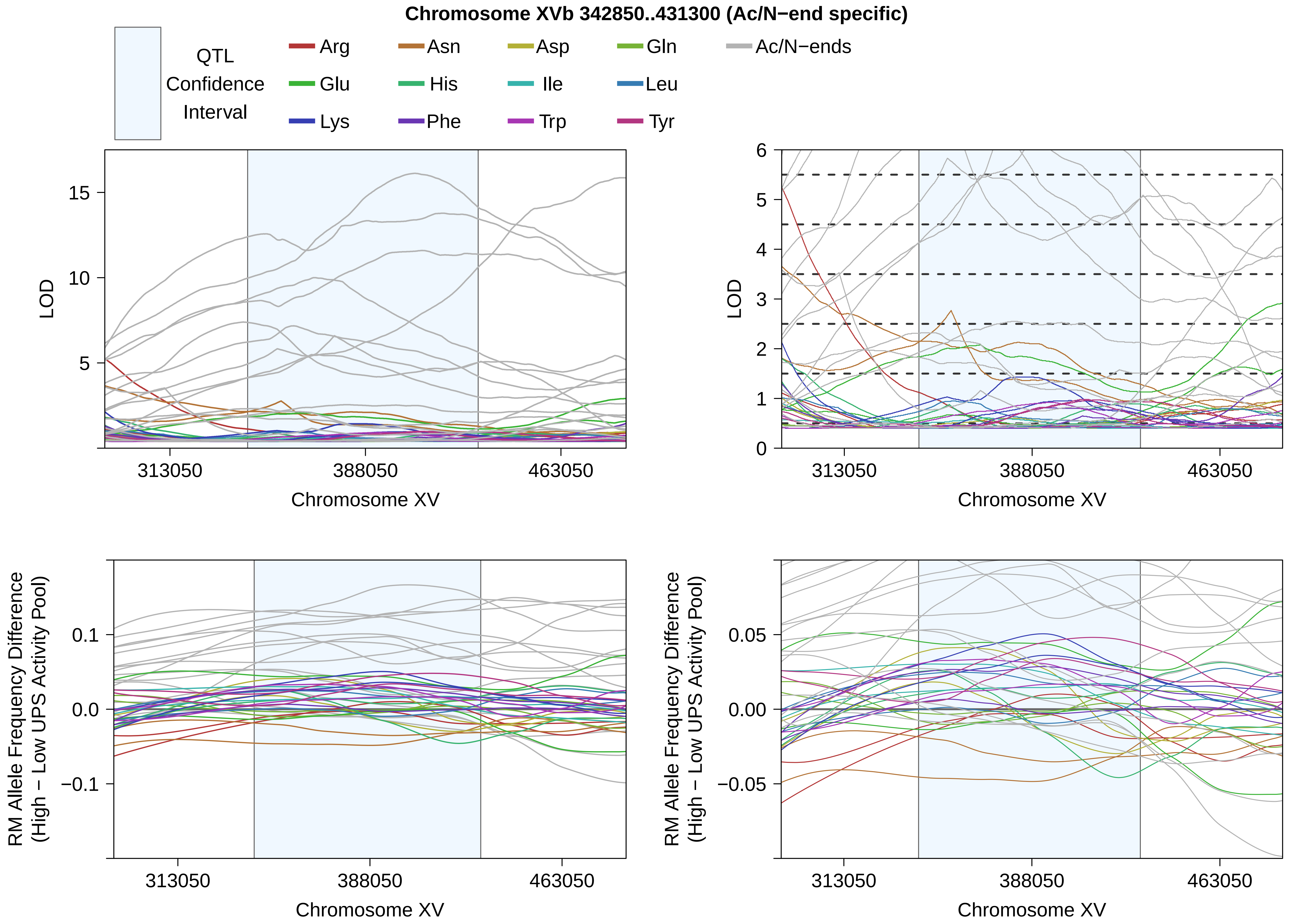
Chromosome XVb 342850..431300 (Ac/N−end specific)
QTL Confidence Interval
Arg
Asn
Asp
Gln
Ac/N−ends
Glu
His
Ile
Leu
Lys
Phe
Trp
Tyr
LOD
Chromosome XV
313050
388050
463050
RM Allele Frequency Difference (High − Low UPS Activity Pool)

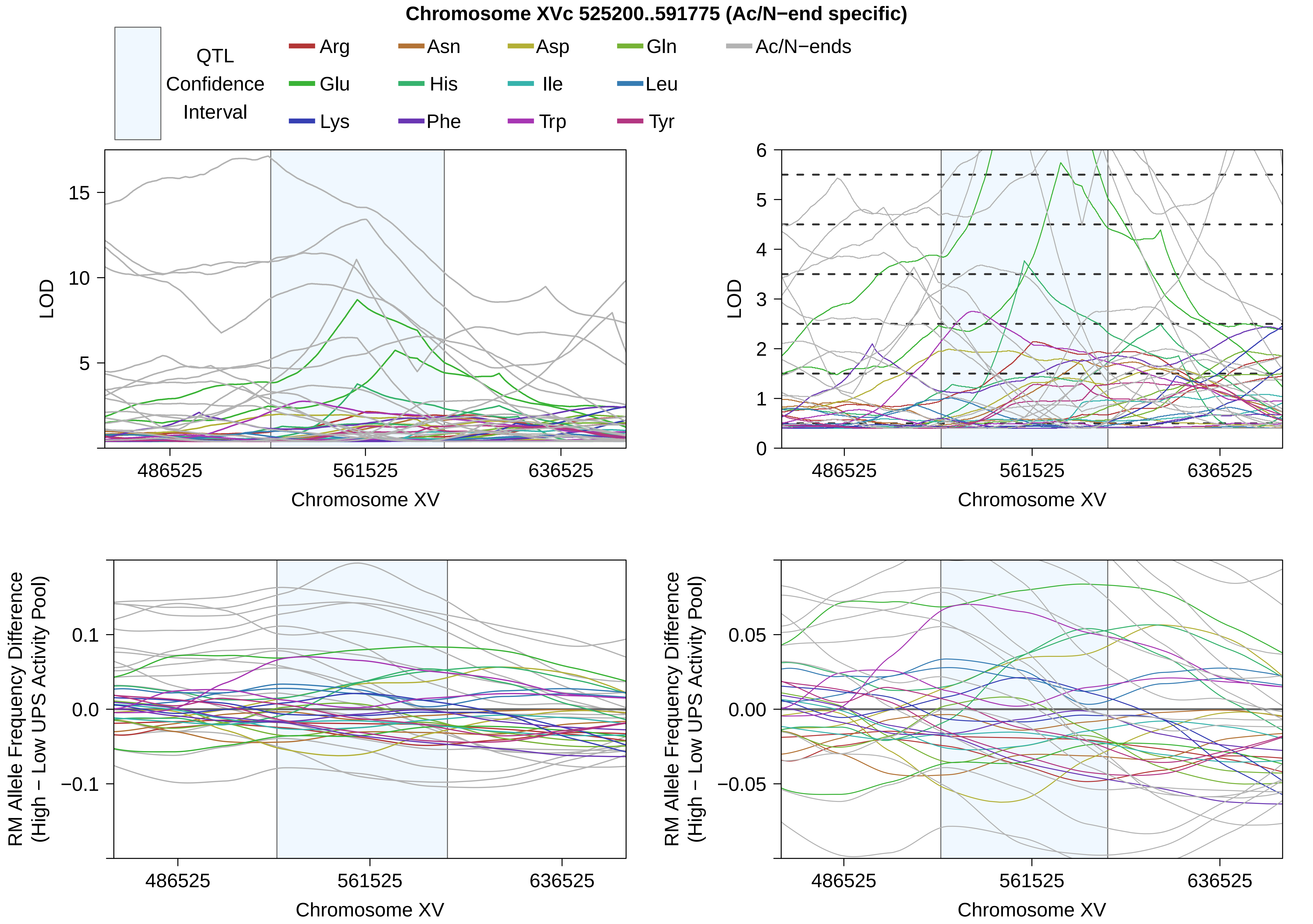

Chromosome XVc 525200..591775 (Ac/N−end specific)
QTL Confidence Interval
Arg
Asn
Asp
Gln
Ac/N−ends
Glu
His
Ile
Leu
Lys
Phe
Trp
Tyr
LOD
Chromosome XV
486525
561525
636525
RM Allele Frequency Difference (High − Low UPS Activity Pool)

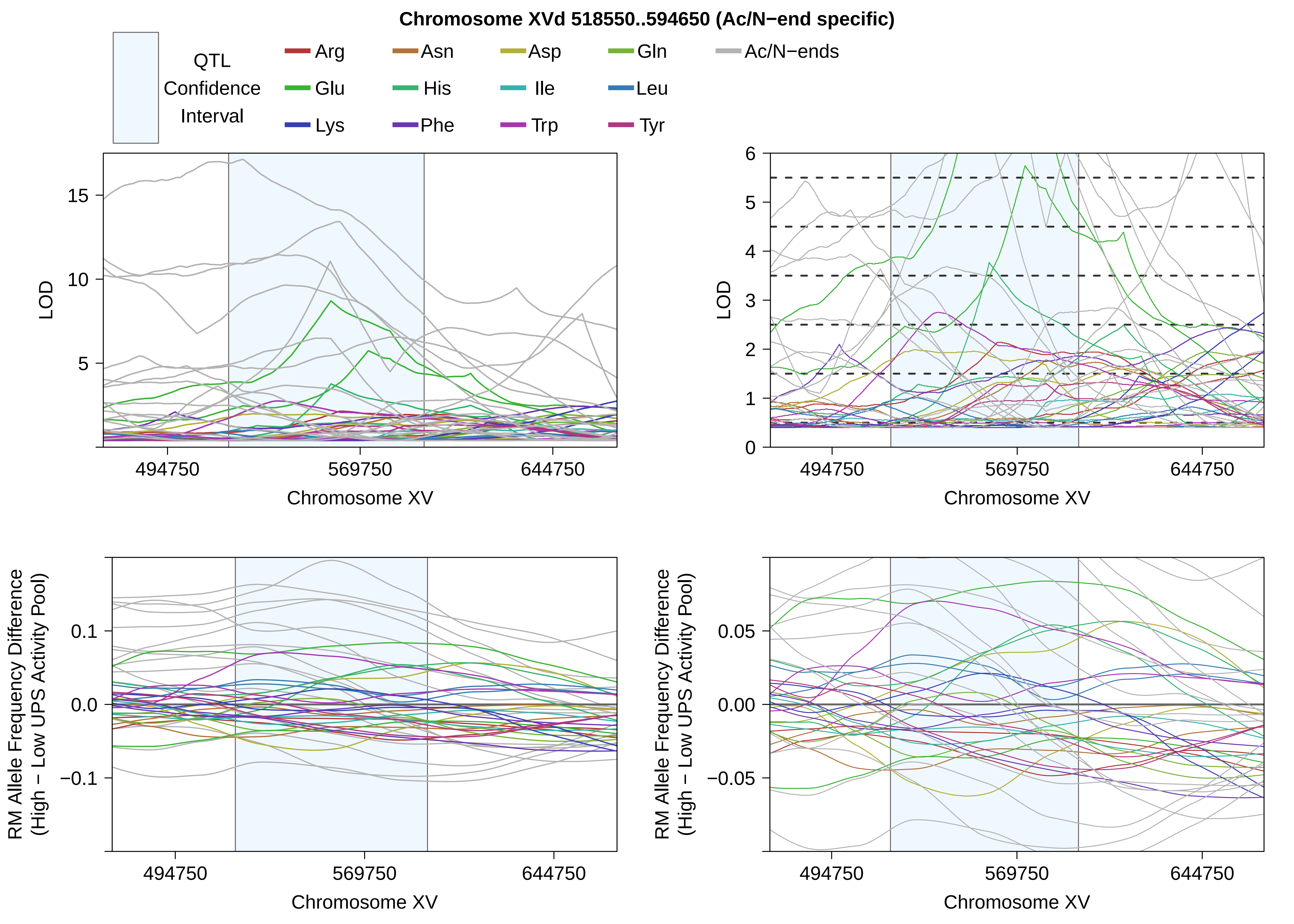
Chromosome XVd 518550..594650 (Ac/N−end specific)
QTL Confidence Interval
Arg
Asn
Asp
Gln
Ac/N−ends
Glu
His
Ile
Leu
Lys
Phe
Trp
Tyr
LOD
Chromosome XV
494750
569750
644750
RM Allele Frequency Difference (High – Low UPS Activity Pool)

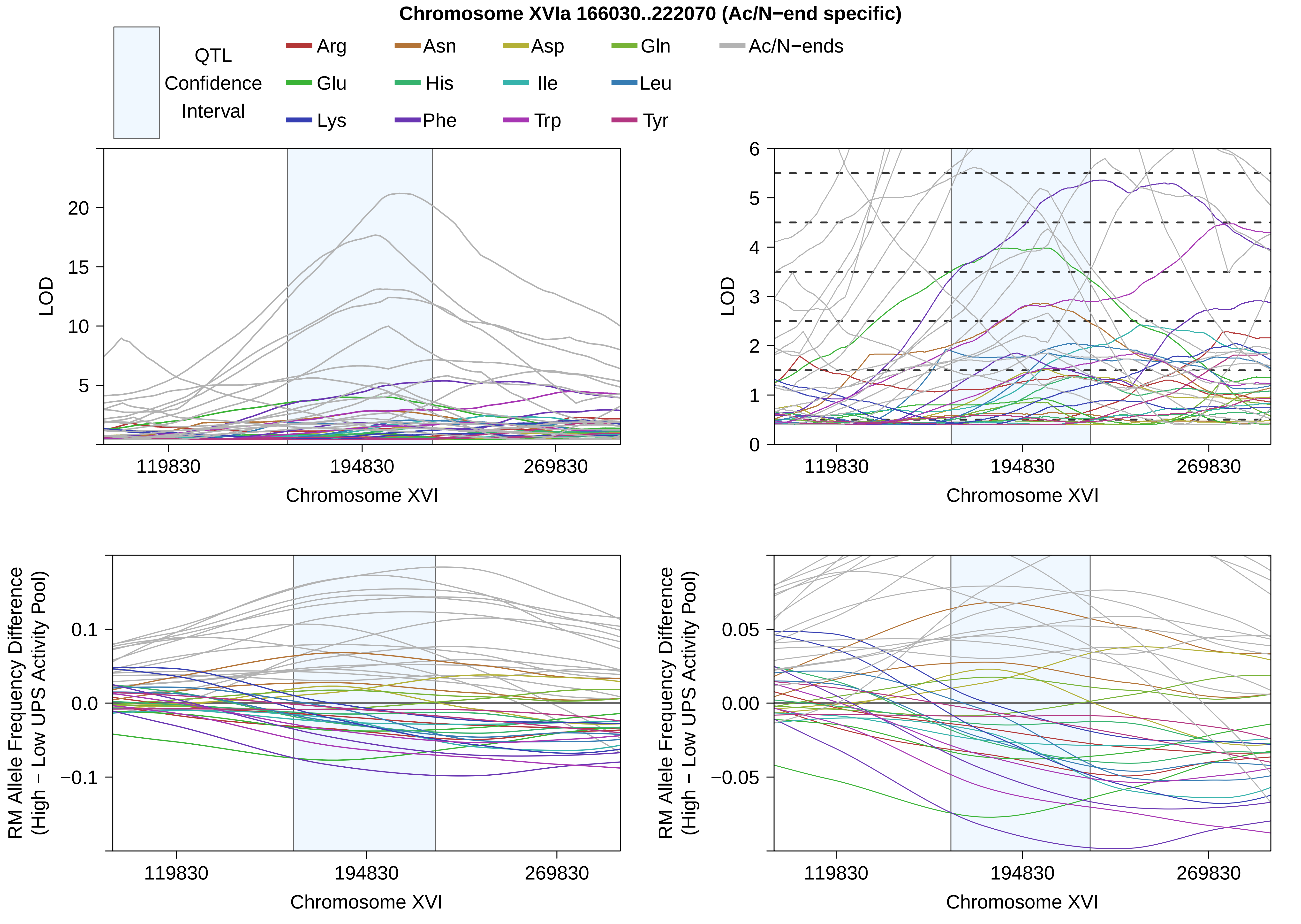
Chromosome XVIa 166030..222070 (Ac/N−end specific)
QTL Confidence Interval
Arg
Asn
Asp
Gln
Ac/N−ends
Glu
His
Ile
Leu
Lys
Phe
Trp
Tyr
LOD
Chromosome XVI
119830
194830
269830
RM Allele Frequency Difference (High – Low UPS Activity Pool)

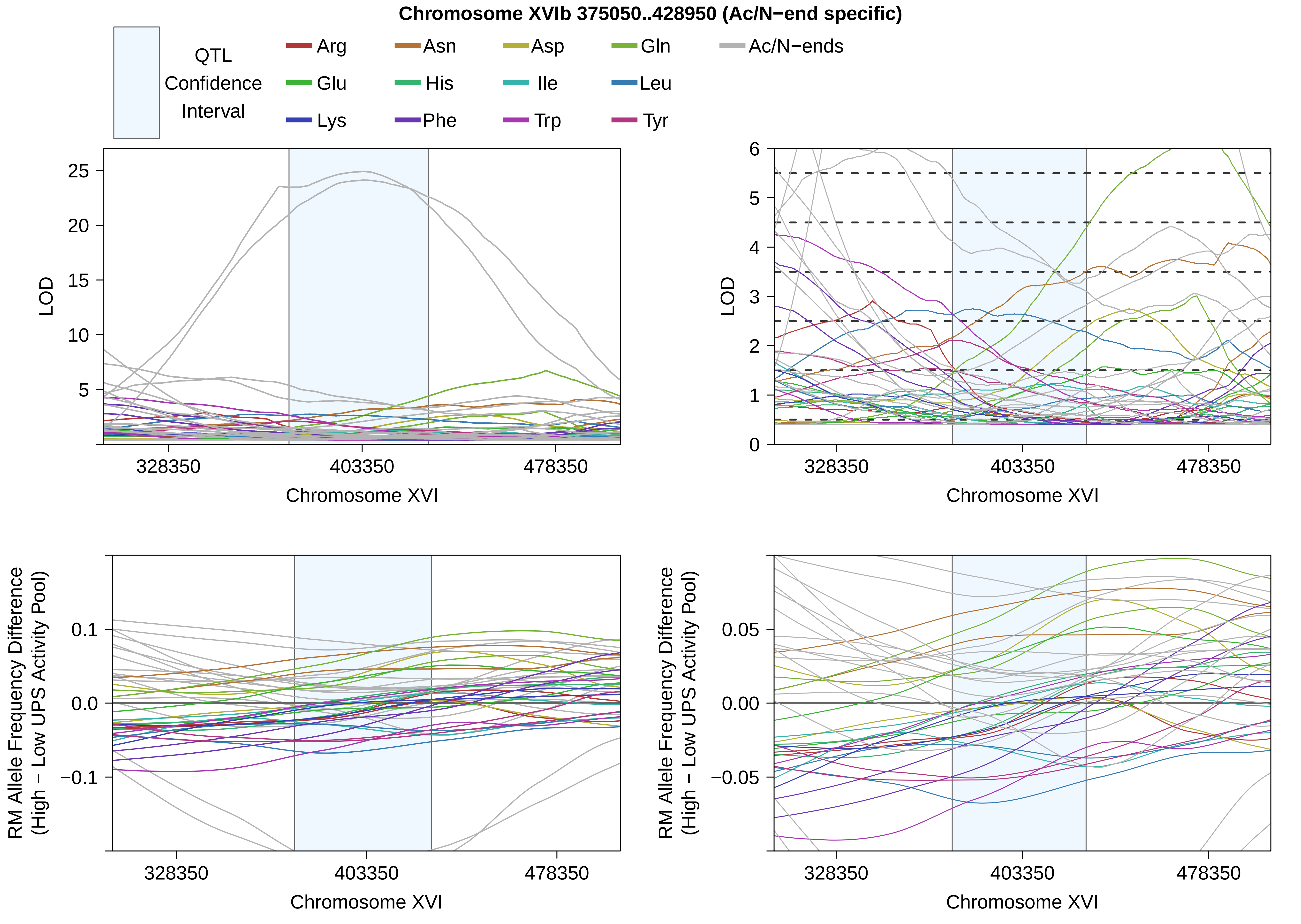

Chromosome XVIb 375050..428950 (Ac/N−end specific)
QTL Confidence Interval
Arg
Asn
Asp
Gln
Ac/N−ends
Glu
His
Ile
Leu
Lys
Phe
Trp
Tyr
LOD
Chromosome XVI
328350
403350
478350
RM Allele Frequency Difference (High − Low UPS Activity Pool)
